# Facile Fabrication of Large‐Area and High‐Quality Organic–Inorganic Hybrid Perovskite Ferroelectric Films Through Electrohydrodynamic Printing

**DOI:** 10.1002/advs.202414122

**Published:** 2025-04-01

**Authors:** Jingjing Luo, Zhongqi Ren, Xue Qi, Qiang Pan, Dong Li, Yuan Xiong, Jie Yao, Haipeng Liu, Suzhu Yu, Jun Wei

**Affiliations:** ^1^ Shenzhen Key Laboratory of Flexible Printed Electronics Technology School of Materials Science and Engineering Harbin Institute of Technology Shenzhen 518055 P. R. China; ^2^ Jiangsu Key Laboratory for Science and Applications of Molecular Ferroelectrics Southeast University Nanjing 211189 P. R. China

**Keywords:** EHD printing, high‐quality, large‐scale, OIHPF film

## Abstract

Traditional oxide ferroelectrics have long struggled to produce flexible, large‐area, and high‐quality thin films at low temperatures, despite their good piezoelectric activity. Organic–inorganic hybrid perovskite ferroelectrics (OIHPFs), by contrast, are gaining popularity as promising candidates for next‐generation flexible self‐powered electric devices due to their easy solution‐processability, structural adjustability, mechanical flexibility, and superior piezoelectric performance. However, their practical use is hindered by a lengthy single‐crystal growing period and the absence of a truly effective large‐area film manufacturing technique. To address these challenges, a simple electrohydrodynamic (EHD) printing technology is first proposed to in situ manufacture large‐area, high‐quality OIHPF crystals or flexible films at low temperatures, without substrate or size limitations. The obtained TMCM‐CdBrCl_2_ film has a high coverage of 99.83%. Notably, it has a crystallinity of 80.35% and a superior piezoelectric coefficient that is 72% and 5.8 times higher than that of the non‐EHD‐printed sample, respectively. The flexible piezoelectric nanogenerator (PENG) based on this film can endure over 200 000 bending cycles—the highest value recorded to date. This approach also shows strong feasibility and broad applicability in creating different OIHPF films or crystals, providing valuable scientific insight for the large‐scale and integrated development of flexible OIHPF‐based electronics.

## Graphical abstract

In situ fabrication of large‐area, high‐quality organic‐inorganic hybrid perovskite ferroelectric (OIHPF) crystals and films via on‐demand electrohydrodynamic (EHD) printing modes.

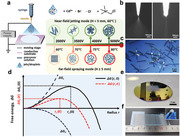

## 1 Introduction

Organic–inorganic hybrid perovskite ferroelectrics (OIHPFs), which combine the benefits of organic and inorganic building blocks, are prominent subsets of molecular ferroelectric materials.^[^ ^1^, ^2^ ^]^ They are widely used in nonvolatile memory devices, actuators, transducers, piezoelectric sensors, and energy harvesting due to their outstanding solution processability, structural tunability, and mechanical flexibility.^[^ ^3^, ^4^ ^]^ More importantly, these ferroelectrics, particularly 1D materials, exhibit piezoelectric properties comparable to oxide‐based ferroelectrics, making them intriguing candidates for lead‐free piezoelectric materials.^[^ ^1^ ^]^ As an example, Xiong's group^[^ ^5^ ^]^ discovered a considerable piezoelectric stain constant d_33_ of 383 pC N^−1^ for 1D trimethyl chloromethylammonium cadmium trichloride (TMCM‐CdCl_3_), which is much higher than that of BaTiO_3_ (220 pC N^−1^). Additionally, Hu et al.^[^ ^6^ ^]^ reported a 1D soft solid solution ferroelectric of C_6_H_5_N(CH_3_)_3_CdBr_2_Cl_0.75_I_0.25_, exhibiting a high d_33_ of 367 pm V^−1^ and low mechanical softness of 0.8 GPa, and it is even lower than that of human hair (2 GPa). As a result, OIHPFs display great potential in the development of lightweight, thin, and flexible self‐powered electronic devices. Wu et al.^[^ ^7^ ^]^ prepared a metal‐free perovskite (MDABCO‐NH_4_I_3_)‐based piezoelectric nanogenerator (PENG), serving as self‐powered strain sensors for applications including human‐machine interfaces or in vitro electrical stimulation devices.

The benefits of OIHPFs mentioned above have attracted a lot of interest expanding their uses beyond traditional ferroelectric and piezoelectric applications to solar cells,^[^ ^8^ ^]^ photodetectors,^[^ ^9^ ^]^ and light‐emitting diodes.^[^ ^10^ ^]^ However, due to the inherent brittleness and rigid growth conditions in single crystal forms,^[^ ^5^, ^11^ ^]^ OIHPF polycrystalline films have become the preferred option for developing flexible electronic devices. Currently, polycrystalline OIHPFs thin film production is limited to simple and rudimentary solution‐based processes like drop‐casting^[^ ^12^ ^]^ and spin‐coating.^[^ ^13^ ^]^ These techniques are scale‐limited and often provide films of questionable quality, replete with defects and low crystallinity, which significantly restricts the potential uses and advancements of OIHPF thin‐film electronic devices. Thus, the development of an easy‐to‐use, effective, and adaptable process for producing high‐quality, large‐area OIHPF films is a must.

In this study, we proposed a facile one‐step “down‐to‐up” preparation technique for in‐situ producing large‐scale OIHPF films based on “Far‐field” electrohydrodynamic (EHD) spray printing. By optimizing the printing and post‐sintering conditions, we obtained high‐quality, dense TMCM‐CdBrCl_2_ films while maintaining their original crystal structure. The EHD's built‐in electric field strength and post‐sintering temperature had a substantial effect on the microstructure, crystallinity, and piezoelectric coefficient of TMCM‐CdBrCl_2_ films. The optimized film demonstrated 99.83% coverage and 80.35% crystallinity, featuring a piezoelectric coefficient that was 5.8 times greater than that of the non‐EHD‐printed (spin‐coating) sample. The film‐based flexible PENG could withstand over 200 000 bending cycles, the most ever documented. The method was also proved to be successful in creating other large‐area OIHPF thin films, such as C_6_H_5_N(CH_3_)_3_CdBr_2_Cl_0.75_I_0.25_, providing an important insight for future large‐scale application of OIHPF films. Besides, by using EHD “Near‐field” jetting mode, the micron‐sized OIHPF crystal grains can be also created in situ, which offers a crucial research avenue for replacing the sluggish solution‐based growing method previously used for generating large‐size OIHPF single crystals.

## 2 Results

### 2.1 Selective Fabrication TMCM‐CdBrCl_2_ Crystal or Film Deposited at Different EHD Printing Modes via Coupling Electric‐Field Effect and Size Effect

**Figure 1:**
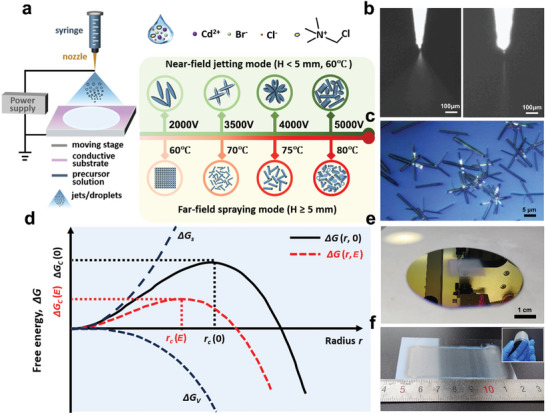
In situ fabrication of TMCM‐CdBrCl_2_ crystal or film: a) Schematic of an EHD printer operating at different modes; b) Actual printing pictures of “Far‐field” spraying mode (left) and “Near‐field” jetting mode (right); c) “Near‐field” jetting mode used to produce TMCM‐CdBrCl_2_ rod‐like crystals on ITO glass; d) Illustration of Δ*G* curves of a growing crystal nucleus as a function of crystal radius w/wo ***EF*** involved; e) Silicon wafer‐supported and f) ITO/PET‐deposited TMCM‐CdBrCl_2_ film prepared using “Far‐field” spraying mode.

The TMCM‐CdBrCl_2_ crystal or film was fabricated using a non‐contact deposition technique via an EHD printer (**Figure** **1**a; Figure S1, Supporting Information), as detailed in Text S1 (Supporting Information). During the printing process, varying electric field (***EF***) forces are generated between the nozzle tip and conductive substrate to overcome the surface tension of TMCM‐CdBrCl_2_ precursor solution composed of rich ions, including TMCM^+^, Cd^2+^, Cl^−^, and Br^−^. When ***EF*** force is large enough or even stronger controlling through the applied voltage (***U***) or nozzle tip‐to‐substrate distance (printing height, ***H*)**, it pulls the fluid to form thin jets or numerous nano‐micro droplets (Figure 1b; Movies S1 and S2, Supporting Information), corresponding to the “Near‐field” jetting or “Far‐field” spraying mode, respectively. As a result, the rod‐like TMCM‐CdBrCl_2_ crystals are produced under the “Near‐field” jetting mode (Figure 1c), which further accumulate into flower‐like clusters with increasing ***EF*** (Figure 1a). Interestingly, the “Far‐field‐sprayed” TMCM‐CdBrCl_2_ appears as nanoparticles first and then changes into rod‐shaped crystals with rising sintering temperature (***T_S_***) (Figure 1a). The detailed crystallization mechanism of the film or crystal is depicted in Figure 1d and will be elaborated upon later with a focus on the coupling effects of ***EF*** and size. As demonstrated in Figure 1e, the “Far‐field‐sprayed” TMCM‐CdBrCl_2_ film exhibits good surface morphology when a conductive substrate, such as a hard silicon wafer, is utilized as the collector. Furthermore, flexible substrates with varying sizes and geometries, such as ITO‐coated PET, can be used to fabricate large‐area TMCM‐CdBrCl_2_ film, thereby proving the effectiveness of EHD‐sprayed films (Figure 1f).

### 2.2 Morphology and Structural Characterizations of EHD‐Printed Crystals or Films

A standard serpentine “S” routing pattern was utilized for the printing trajectory, as depicted in Figure S2 (Supporting Information) and elaborated upon in Text S2 (Supporting Information). To investigate the impact of ***EF*** on the morphology of EHD‐printed TMCM‐CdBrCl_2_ during actual printing, a working window of SEM images defined by ***U*** and ***H*** was established, as illustrated in Figure S3 (Supporting Information). The window was devised considering practical printing parameters and safety constraints as detailed in Text S3 (Supporting Information), with ***U*** ranging from 2000 to 6000 V, and ***H*** spanning from 3 to 9 mm. Additional parameters, including nozzle tip diameter (***D***), air pressure (***P***, generated by an air compressor, as seen in Figure S1, Supporting Information), line distance (***d***), stage moving speed (***v***), and ***T_S_***, were maintained constant at 35 µm, 0 kPa, 0.25 mm, 2 mm s^−1^, 60 °C, respectively. Notably, two distinct trends were observed. One trend involved the TMCM‐CdBrCl_2_ film prepared with ***H*** exceeding 3 mm and ***U*** below 6000 V (indicated by the interwoven blue and yellow lines), which exhibited stacked nanoparticles, corresponding to the “Far‐field” spraying mode. The other trend exhibited isolated or aggregated rod‐like crystals with substantial lengths of several micrometers, associated with the “Near‐field” jetting mode. The findings demonstrate that the ***EF*** strength, governed by ***U*** and ***H***, exerts a substantial influence on the morphology and dimensions of TMCM‐CdBrCl_2_, resulting in two distinct EHD printing modes.

To elucidate the distinctive deposition process of TMCM‐CdBrCl_2_ crystals or films, the coupling effects of ***EF*** and size are proposed, grounded in the intrinsic ***EF*** and non‐contact printing feature of EHD technology. The ***EF*** is pivotal in facilitating the successful implementation of EHD printing, inducing the formation of thin jets or a multitude of nano‐ to micro‐sized droplets. The non‐contact nature of EHD printing allows for the formation of these jets or droplets, which can be effectively explained by classical homogeneous nucleation theory, owing to the lack of preferential nucleation sites inherent in EHD printing.^[^ ^14^, ^15^, ^16^ ^]^ For simplicity, a single droplet is considered as a spherical particle, its total free energy Δ ***G*** (Figure 1d) is equal to the sum of the negative bulk free energy Δ ***G_V_*** and positive surface free energy Δ ***G_S_***, as defined using thermodynamic Equation (1).

(1) $$\Delta G\;\left(r,0\right)=\Delta G_{S}+\Delta \;G_{V}=4\pi r^{2}\gamma -\frac{4}{3}\pi r^{3}A\ln S$$

(2) $$\left(A=\frac{\rho \mathrm{RT}}{M},R=N_{A}k_{B}\right)$$

where ***r***, ***γ***, ***A***, and ***S*** are the radius of spherical particles, solution‐crystal interfacial energy, pre‐exponential factor, and the degree of supersaturation, while ***ρ***, ***R***, ***T***, and ***M*** represent the mass density, universal gas constant, absolute temperature and molar mass of the solid, and ***N_A_*** and ***k_B_*** refer to the Avogadro's number and Boltzmann constant, respectively.

When ***EF*** is introduced, Equation (1) can be updated as given by^[^ ^17^ ^]^

(3) $$\Delta G\;\left(r,E\right)=\Delta G_{S}+\Delta \;G_{V}=4\pi r^{2}\gamma -\frac{4}{3}\pi r^{3}\left(\mathrm{AlnS}+aE^{2}\right)$$

here, ***E*** denotes the electric field strength, **a** is a constant related to ***E***, and the additional free energy is taken into account from electrostatic free energy in contrast to Equation (1). The critical nuclei radius ***r_c_*** referring to the free‐energy barrier to nucleation with or without ***EF*** can be stated by

(4) $$r_{c}(0)=\frac{2\gamma }{A\ln S}\;$$

(5) $$r_{c}(E)=\frac{2\gamma }{A\ln S+aE^{2}}\;$$

Obviously, the existence of ***EF*** significantly lowers the critical cluster radius (***r_c_*** (*E*) < ***r_c_*** (*0*)), indicating that those particles with a radius in the range of (***r_c_*** (*E*), ***r_c_*** (*0*)) will enter supercritical zone early to nucleate. Accordingly, the nucleation rate involved with ***EF*** is improved according to Equation (6) and (7) as

(6) $$\frac{\mathrm{dN}}{\mathrm{dt}}\left(0\right)=A\exp\left[\frac{-\Delta G_{N}}{k_{B}T}\right]=A\exp\left[-\frac{16\pi y^{3}}{3k_{B}T{\left(A\ln S\right)}^{2}}\right]$$

(7) $$\frac{dN}{dt}\left(E\right)A\exp\left[\frac{-\Delta G_{N}}{k_{B}T}\right]=A\exp\left[-\frac{16\pi y^{3}}{3k_{B}T{\left(A\ln S+aE^{2}\right)}^{2}}\right]$$

where ***N*** is the number of nuclei. It is readily apparent that the nucleation rate is positively correlated with ***S***, ***T***, and ***E***. Of these factors, ***E*** is deemed the primary determinant due to its second‐order exponential influence, signifying the ***EF*** effect. Conversely, the influence of ***S*** is attributed to the reduction in ***r***, which is categorized as the size effect.

The preceding explanation aptly elucidates how the coupling effects of ***EF*** and size theoretically promote nucleation. However, their dominance varies in the practical printing process. On the one hand, ***EF*** is intensely concentrated near the nozzle, where positively charged cations preferentially accumulate at the tip, while others distribute internally to maintain charge equilibrium^[^ ^18^ ^]^ (as shown in Figure S2, Supporting Information). When thin jets or nano‐micro droplets are propelled into the air, solvent evaporation accelerates due to the size effect, resulting in an increased surface‐area‐to‐volume ratio of the airborne droplets.^[^ ^19^ ^]^ The coupling effects enhance the local supersaturation of TMCM‐CdBrCl_2_ precursor solution prior to its arrival at the printing substrate, culminating in the in‐situ crystallization of rods or nanoparticles. On the other hand, as observed in Figure S3 (Supporting Information), rod‐like crystals tend to form under the EHD “Near‐field” jetting mode, which is predominantly influenced by a strong ***EF*** effect. Conversely, nanoparticles are generated under the “Far‐field” spraying mode, where the size effect becomes more pronounced due to the relatively moderate ***EF*** strength.

A comprehensive analysis of the morphological evolution of TMCM‐CdBrCl_2_ under various EHD printing modes is detailed in Text S4 (Supporting Information). Considering the overall quality of the fabricated films, the film prepared at an ***H*** of 7 mm and a ***U*** of 3500 V (referred to as 7–3500) is considered optimal, demonstrating excellent coverage and surface flatness. Nonetheless, the film appears insufficiently dense (as illustrated in Figure S4a, Supporting Information).

**Figure 2:**
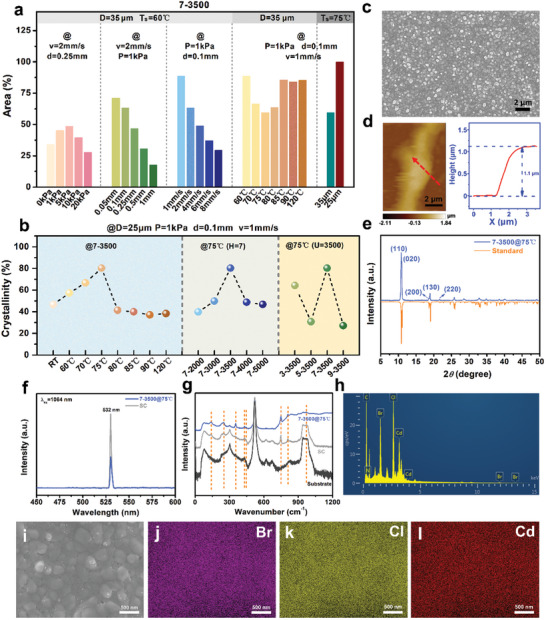
EHD‐sprayed TMCM‐BrCdCl_2_ films prepared under different printing parameters: Statistical results of a) crystal coverage and b) crystallinity. The optimal TMCM‐CdBrCl_2_ film: c) SEM image; d) Thickness measured by decreased numbers of print lines; e) PXRD pattern in comparison with the standard one; f) SHG spectra; g) Raman spectra; and h–l) EDS element mapping results.

Under the “Far‐field” spraying mode at 7–3500, the film quality is further optimized by adjusting variables such as ***P***, ***d***, and ***v*** through a single‐factor approach, as depicted in Figure S4 (Supporting Information). During the process, Image J software is utilized to statistically analyze crystal coverage. As illustrated in **Figure** **2**a, film coverage initially increases and then decreases with rising ***P***, peaking at 48.54% at 5 kPa. However, high ***P*** induces turbulence among the droplets, thereby reducing coverage. To ensure long‐term printing stability, a low pressure of 1 kPa was employed at 7–3500 for subsequent experiments. Additionally, a similar trend was observed with increases in ***d*** and ***v***, where larger values of ***d*** or ***v*** result in smaller crystal grains, adversely impacting film coverage. Excessively small ***d*** leads to particle aggregation, resulting in crystal flocculates that diminish the film's flatness. Following a comprehensive evaluation of the film's coverage and surface flatness, the optimal parameters were determined to be *P* = 1 kPa, *d* = 0.1 mm, and *v* = 1 mm s^−1^. Under these conditions, the crystal coverage reached a peak of 88.57%, indicating the presence of residual nanopores within the film.

To address this, alternative strategies such as increasing the number of printing layers, adjusting ***T_S_***, and modifying ***D*** were explored to fabricate the desired TMCM‐CdBrCl_2_ film. However, increasing the number of printing layers did not produce satisfactory results. As depicted in Figure S5a–c (Supporting Information), multi‐layer printing encourages crystal grain growth in both the X‐Y plane and Z‐axis, thereby failing to achieve a dense film. This phenomenon becomes more pronounced with an increasing number of layers and can be attributed to the selective growth along the polarization direction characteristic of TMCM‐CdBrCl_2_.^[^ ^20^ ^]^ As previously mentioned, the EHD spraying mode induces the formation of small droplets, leading to rapid saturation and nucleation due to the size effect, which likely results in the imperfect crystallization of TMCM‐CdBrCl_2_. Consequently, crystals continue to grow with increased packing density as the number of printing layers increases.

To enhance film coverage, a post‐sintering procedure based on single‐layer printing was selected for the subsequent studies. As demonstrated in Figure S5d–i (Supporting Information), higher ***T_S_***, initially causes granular crystals to melt and expand, forming large‐size crystals. At ≈75 °C, TMCM‐CdBrCl_2_ nanoparticles fully evolve into a rod‐shaped interwoven structure. Subsequently, the rod‐like structure disappears and reverts to granular crystals. We hypothesize that the temperature‐induced changes in crystal morphology may be associated with the phase transition in EHD‐sprayed TMCM‐CdBrCl_2_. To investigate this, DSC and temperature‐dependent dielectric measurements were performed, with the results illustrated in Figure S6 (Supporting Information). Prior to this, the thermal stability of TMCM‐CdBrCl_2_ was assessed (Figure S7, Supporting Information), indicating that the material remains stable up to 517 K. The combined DSC and dielectric data reveal that the printed TMCM‐CdBrCl_2_ experiences thermal anomalies ≈350 and 368 K, respectively. Although the initial phase transition peak in the DSC heating curve is not well‐defined, it can be attributed to the rapid heating rate during measurement. Additionally, the transition of TMCM‐CdBrCl_2_ from a granular morphology to oriented, rod‐like crystals in AFM image was distinctly observed in the film sintered at 75°C for a short duration of 6 h (Figure S8, Supporting Information). This further confirms that the TMCM‐CdBrCl_2_ nanoparticles are prone to melting and regrowth near the phase transition temperature, ultimately forming their intrinsic 1D rod‐like crystal structure.

To ascertain the crystal structure changes in these ***U***, ***H***, or ***T***‐induced films, PXRD measurements were conducted, as shown in Figure S9 (Supporting Information). The results indicate that all samples in this study are in excellent concordance with the previous report,^[^ ^20^ ^]^ with the sample labeled ‘Standard’ serving as the reference. The crystallinity result obtained via the Origin “curve‐fitting” method is presented in Figure 2b. The TMCM‐CdBrCl_2_ film sintered at 75 °C exhibited a maximum crystallinity of ≈80.35%, which is 72% higher than that of the sample at ambient temperature. Excessively high temperatures, however, may hinder crystallization, as seen in Figure S5 (Supporting Information).

The aforementioned data provides compelling evidence that a post‐sintering treatment around the phase transition temperature facilitates a substantial enhancement in the crystallinity of TMCM‐CdBrCl_2_. However, as Figure 2a illustrates, the objective of producing high crystal coverage film is hindered by the trade‐off, where the increase in crystallinity through high‐temperature sintering compromises the film's crystal coverage. This finding motivated us to enhance the film density further by optimizing the parameter ***D***. As shown in Figure S5j–l (Supporting Information), the crystals exhibited a 3D fibrous aggregation structure when ***D*** was reduced to 15 µm. Constructing a microscopically dense film proved challenging when ***D*** was increased to 45 µm, as large TMCM‐CdBrCl_2_ rod‐like crystals and background nanoparticles appeared simultaneously. Alternatively, we fabricated a high‐quality TMCM‐CdBrCl_2_ film using a 25 µm nozzle, achieving a microscopically dense structure (Figure S5k, Supporting Information) and a macroscopically flat surface (Figure 2c).

Based on these findings, *D* = 25 µm, 7–3500@75 °C, *P* = 1 kPa, *d* = 0.1 mm, and *v* = 1 mm s^−1^ were identified as the optimal printing parameters for producing a high‐quality TMCM‐CdBrCl_2_ film. The resulting film is ≈1.1 µm thick (Figure 2d) and exhibits an exceptional crystal coverage of up to 99.83% (Figure 2a). For comparison, the spin‐coated TMCM‐CdBrCl_2_ films were prepared by varying the speed or concentration of the precursor solution (Figures S10 and S11, Supporting Information). However, all attempts failed due to poor surface coverage.

Notably, Figure 2e; Figure S12 (Supporting Information) illustrate that the crystal structure of TMCM‐CdBrCl_2_ was preserved throughout the printing process,^[^ ^20^ ^]^ corroborated by the PXRD Rietveld refinement results shown in Figure S13 (Supporting Information). The analysis conducted using GSAS II revealed excellent concordance between the observed and calculated diffraction patterns. The Rietveld refinement confirmed that the sample adopts the monoclinic space group Cc (No. 9). The refined lattice parameters were established as ***a*** = 9.5175(17) Å, ***b*** = 16.1187(21) Å, ***c*** = 6.8887(9) Å, and ***β*** = 93.168°. The residual factors, ***R_wp_*** and ***R_p_***, were 5.76 and 4.65, respectively, indicating a superior fit and underscoring the accuracy of the structural model. Moreover, SHG measurements of both the spin‐coated (3500 rpm, 30 s) and EHD‐sprayed films revealed prominent peaks (Figure 2f), unequivocally confirming the material's non‐centrosymmetric nature‐a hallmark of ferroelectric and piezoelectric materials.^[^ ^21^ ^]^ Furthermore, the Raman spectra of the two films displayed identical peak positions, suggesting that films produced using these distinct fabrication methods possess comparable crystalline structures, as seen in Figure 2g. Specifically, the band observed at 143 cm^−1^ corresponds to the rotation motion of the Cd(ClBr)_6_ octahedron. The peaks detected at 252, 352, 431, and 446 cm^−1^ are attributed to the symmetric and asymmetric stretching vibrations of the Cd─Cl and Cd─Br bonds.^[^ ^22^ ^]^ The band appearing at 750 cm^−1^ is associated with the symmetric and asymmetric stretching vibrations of the C─N bond. The peaks identified at 810 and 970 cm^−1^ correspond to the out‐of‐plane deformation vibrations of C─H bonds.^[^ ^22^ ^]^ EDS scanning results of the EHD‐sprayed film, depicted in Figure 2 h‐l, revealed a uniform distribution of the characteristic organic and inorganic elements Br, Cl, and Cd. Comparable results were observed for the rod‐like crystal structures (Figure S14, Supporting Information), further affirming that the crystal materials fabricated via EHD printing align with our anticipated specifications.

Expanding upon this research strategy, the technique was further refined to fabricate a range of large‐area OIHPF thin films and crystal materials, including C_6_H_5_N(CH_3_)_3_CdBr_2_Cl_0.75_I_0.25_. The film was produced with the following optimized parameters: *D* = 25 µm, 5–4000@70 °C, *v* = 1 mm s^−1^, *P* = 1 kPa, and *d* = 0.25 mm for a single layer. As depicted in Figure S15 (Supporting Information), dense C_6_H_5_N(CH_3_)_3_CdBr_2_Cl_0.75_I_0.25_ thin film was successfully fabricated using the “Far‐field” spraying mode (Figure S15a, Supporting Information), achieving a thickness of ≈108 nm (Figure S15b, Supporting Information). Figure S15c (Supporting Information) illustrates that the PXRD diffraction peaks of the resulting thin film aligned well with the standard pattern,^[^ ^6^ ^]^ except for several peaks showing significant orientation and shift. SHG measurements (Figure S15d, Supporting Information) were conducted to confirm the spatial symmetry breaking observed in both spin‐coated and EHD‐printed films.^[^ ^23^ ^]^ Additionally, C_6_H_5_N(CH_3_)_3_CdBr_2_Cl_0.75_I_0.25_ crystals with regular large‐size were successfully fabricated on ITO glass using the “Near‐field” jetting mode (Figure S16, Supporting Information).

### 2.3 Ferroelectric and Piezoelectric Analysis

**Figure 3:**
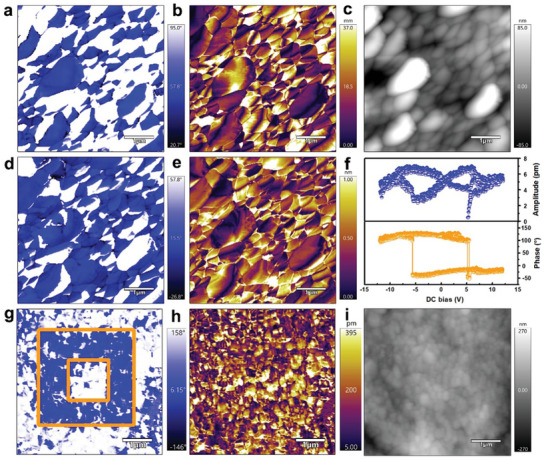
PFM measurements of TMCM‐CdBrCl_2_ film: a,b) Lateral PFM phase and amplitude images, respectively; c) Topo‐graphic image; d,e) Vertical PFM phase and amplitude images, respectively; f) Phase−voltage hysteresis loops and amplitude−voltage butterfly loops obtained with SS‐PFM; g–i) PFM switching spectroscopy measurements: Vertical PFM phase, amplitude, and corresponding topography images. Following poling in the larger blue box region with a tip voltage of +14 V and the smaller blue box region with −14 V, respectively.

The ferroelectric and piezoelectric properties of the TMCM‐CdBrCl_2_ film, including domain structure and switching behavior, were investigated using the non‐destructive PFM technique, as illustrated in **Figure** **3**. PFM images (Figure 3a–e), obtained in both in‐plane (IP) and out‐of‐plane (OP) modes, reveal abundant domain structures, albeit less pronounced in the OP mode. The observed abundance of domain structures is attributed to the multiaxial characteristics of the TMCM‐CdBrCl_2_ film.^[^ ^20^ ^]^ The distinct phase differences, domain walls, and the intriguing “half‐domains” collectively indicate the existence of room‐temperature ferroelectricity in the film. To confirm the macroscopic ferroelectricity of EHD‐printed TMCM‐CdBrCl_2_, ***P***‐***E*** hysteresis loop measurement was performed on a compacted pellet, as shown in Figure S17 (Supporting Information). The measured polarization value of ≈0.054 µC cm^−2^, significantly lower than that of the single crystal reported previously,^[^ ^20^ ^]^ aligns with expectations due to the random orientation of grains in polycrystalline materials. Additionally, PFM measurements of phase and amplitude dependence on DC bias at a selected point (Figure 3f) revealed a hysteresis loop and a characteristic butterfly‐shaped curve, respectively, which are indicative of switchable polarization in the TMCM‐CdBrCl_2_ film. A detailed analysis of domain switching was subsequently performed (Figure 3g–i), revealing a distinctive “box‐in‐box” domain pattern accompanied by a 180° phase shift under an applied external voltage of ±14 V. This observation unequivocally validates the presence of ferroelectric domains.

The TMCM‐CdBrCl_2_ film, prepared with reduced sintering time, was analyzed using PFM to validate the authenticity of the previously mentioned domain signals. Both rod‐like and particle‐like crystal morphologies were identified during the analysis, as illustrated in Figure S18 (Supporting Information). The results reveal the distinct “half‐domain” structures, particularly evident in the rod‐like crystals. In contrast, the film fabricated using the conventional spin‐coating method (Figure S19, Supporting Information) exhibited a random and irregular domain structure, attributed to rapid solvent evaporation and crystallization of TMCM‐CdBrCl_2_. It is plausible to deduce that the distinct domains induced by the applied ***EF*** during EHD printing likely enhance the piezoelectric performance of the films. This enhancement can be attributed to the improved polarization arising from better alignment of TMCM‐CdBrCl_2_ molecular dipoles, which aligns with previously reported findings.^[^ ^24^, ^25^ ^]^

**Figure 4:**
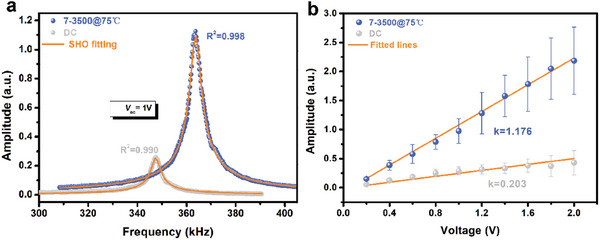
Corrected OP piezoresponse of TMCM‐CdBrCl_2_ film: a) Amplitude versus driving frequency measured under AC drive voltage of 1 V, fitting by SHO model; b) Derived amplitude curve as a function of AC drive voltages.

The local piezoresponse of TMCM‐CdBrCl_2_ films was assessed using the well‐established technique of PFM tip‐sample resonance frequency fitting with a harmonic oscillator (SHO) model. As shown in **Figure** **4**a, the corrected amplitude signals normalized by the Q factor, with ***V_ac_*** (driving AC voltage) set to 1 V, exhibited an excellent fit with the SHO model, verifying the intrinsic piezoresponse of EHD‐sprayed and DC (drop‐coated) TMCM‐CdBrCl_2_ film. For each sample, the average corrected amplitude was measured at ten distinct driving voltage points ranging from 0.2 to 2 V. The slope of the resulting curve represents the relative magnitude of the piezoelectric constant, and the relationship between the corrected amplitude and driving voltage exhibits linearity. As depicted in Figure 4b, the curves display excellent linearity, with a slope of 7–3500@75 °C nearly 5.8 times greater than that of the DC (***EF***‐absent) sample. Subsequently, the effects of parameters ***U***, ***H***, and ***T*** on the piezoresponse of TMCM‐CdBrCl_2_ films were systematically investigated, as illustrated in Figure S20 (Supporting Information). The investigation revealed that with increasing ***U***, ***H***, or ***T***, the piezoelectric response exhibited an initial increase followed by a subsequent decline, reaching its peak at 7–3500@75 °C. These findings provide compelling evidence that optimal ***EF*** and ***Ts*** parameters can significantly enhance the piezoelectric properties of TMCM‐CdBrCl_2_. Additionally, the macroscopic piezoelectric coefficient (d_33_) of the TMCM‐CdBrCl_2_ polycrystalline sample fabricated using EHD printing was evaluated. The compacted pellet, produced through hot pressing followed by polarization treatment, was analyzed using a Berlincourt piezoelectric analyzer. The resulting d_33_ value of 16.9 pC/N is shown in Figure S21 (Supporting Information).

### 2.4 Piezoelectric Performance

**Figure 5:**
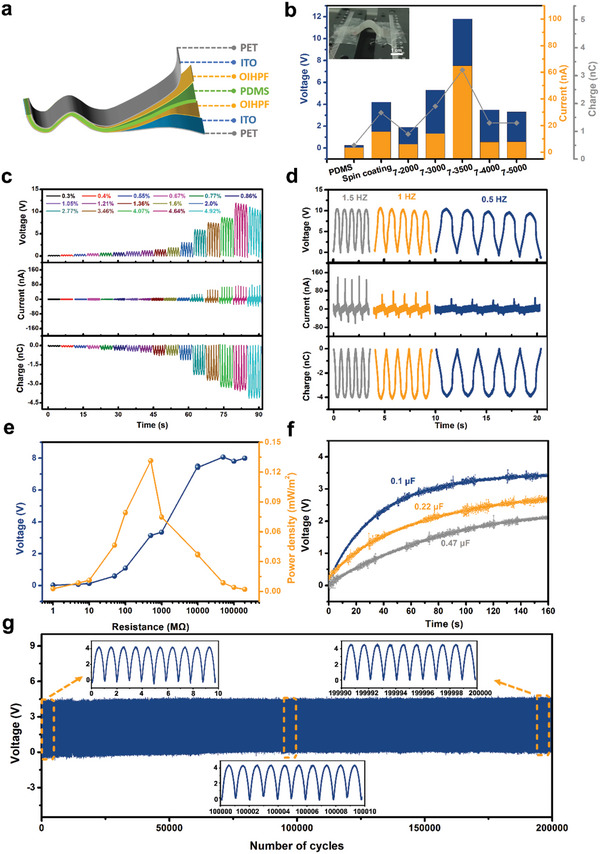
Fabrication and characterizations of TMCM‐CdBrCl_2_ film‐based PENG: a) Schematic illustration of the structure of PENG; b) Piezoelectric outputs of TMCM‐CdBrCl_2_ films prepared by different conditions with a bending strain of 4.64% at 1 Hz. The insert picture in b) reveals the real bending state of 7–3500@75 °C PENG; c) Strain‐ and d) frequency‐dependent piezoelectric output performances of 7–3500@75 °C film when set the frequency and strain as 1 Hz and 4.92%, respectively. e) Calculated output voltage and power density as a function of resistance at 1 Hz. f) Capacitors with varying volumes are charged using the device. g) Output voltage under 200 000 cycles at 3.46% and 1 Hz (insets: waveforms at beginning, middle, and end of cycles).

In order to evaluate the feasibility of piezoelectric TMCM‐CdBrCl_2_ film in real‐life situations, a standard sandwich‐structured piezoelectric nanogenerator (PENG) device was constructed, in which the PDMS layer serves as an intermediate adhesive layer as well as passivation layer.^[^ ^26^ ^]^ **Figure** **5**a provides an exploded view of the TMCM‐CdBrCl_2_‐based PENG. TMCM‐CdBrCl_2_ films made by varying printing voltages were first examined for their piezoelectric outputs at a fixed bending strain of 4.64%. According to Figure 5b, the 7–3500@75 °C film exhibited the best piezoelectric performance, ≈280% higher than that of the spin‐coated film. This outcome is reasonable due to the self‐polarizing nature of the EHD‐printed film, which contrasts with the non‐poled spin‐coated device, thus highlighting the technological advantage of EHD printing. The pristine PDMS film worked as a reference sample to demonstrate the usefulness of the data, with almost negligible response of low open voltage of 0.25 V and short circuit current of 3.5 nA. A switched polarity test of 7–3500@75 °C‐based PENG was also performed. As shown in Figure S22 (Supporting Information), its output signals exhibit opposite polarities but identical magnitudes with forward and reverse connections. The results offer more evidence that the output signal originates from the intrinsic piezoelectricity of the TMCM‐CdBrCl_2_.

A set of bending experiments was conducted to investigate the potential of 7–3500@75 °C in flexible applications. The film's strong strain‐dependent piezoresponse is demonstrated by its increased output signals when bending strains rise, as illustrated in Figure 5c. Moreover, different frequencies were applied to study the impact of frequency on its output signal. According to the results presented in Figure 5d, the film's output voltages and charges stay stable at various frequencies, while there is a positive correlation between higher bending strains and output currents. The result is similar to the previous reports.^[^ ^7^ ^]^ Figure 5e shows the output voltage and power density of theTMCM‐CdBrCl_2_‐based PENG using varying external resistances at a strain of 2.77%. The PENG can achieve a peak power density of 0.13 mW m^−2^ with an external load of 500 MΩ. Figure 5f shows how the device charges various capacitors with a frequency of 1 Hz and load resistance of 500 MΩ. During the charging process, the output voltage gradually rises and eventually reaches saturation, and thus small capacitors are easier to charge and produce greater voltages given their small capacity and low leakage.^[^ ^27^ ^]^ Figure 5g demonstrates the excellent stability of pole‐free TMCM‐CdBrCl_2_‐PENG under a high bending strain of 3.46% for 200 000 cycles. Its output voltage remains almost constant, demonstrating its strong mechanical stability and endurance. This test result is practically the best among all the reports,^[^ ^7^, ^26^, ^28^, ^29^, ^30^, ^31^, ^32^ ^]^ as proved in Table S1 (Supporting Information), indicating the excellent durability of OIHPF film prepared using EHD printing.

Using the same device construction, we examined the use of C_6_H_5_N(CH_3_)_3_CdBr_2_Cl_0.75_I_0.25_‐based PENG as a pressure sensor, as seen in Figure S23 (Supporting Information). The device was first tested for stress‐voltage output dependency (Figure S23a, Supporting Information), in which its output voltage improves with increasing stress but saturates after 50 kg. This suggests that C_6_H_5_N(CH_3_)_3_CdBr_2_Cl_0.75_I_0.25_ has good stress response as a piezoelectric active layer within certain stress. Further sensitivity research, as illustrated in Figure S23b (Supporting Information), demonstrates that it has a segmented linear sensitivity. The sensitivity initially reaches 0.78 V kPa^−1^ within 20.83 kPa, and it subsequently drops to 0.25 V kPa^−1^ between 20.83 and 41.67 kPa. As compared in Table S1 (Supporting Information), the sensitivity with a wide detection range is much superior to that of conventional piezoelectric pressure sensors^[^ ^32^, ^33^, ^34^, ^35^ ^]^ due to the inherently high piezoelectric coefficient of C_6_H_5_N(CH_3_)_3_CdBr_2_Cl_0.75_I_0.25_. Based on this, we conducted the stress limit detection, as shown in Figure S23c (Supporting Information), and determined that the sensor's least detectable stress is 20 Pa. As a pressure sensor, response time is equally important. Figure S23d (Supporting Information) demonstrates that this thin‐film sensor has a response time of 32 ms and a recovery time of 120 ms. As for its frequency response, Figures S23e,f, and S24 (Supporting Information) showed that the film‐based piezoelectric sensor shows frequency dependency in current output but saturation characteristics in voltage and charge outputs, which is similar to prior test results of TMCM‐CdBrCl_2_ strain sensor. Additionally, we also performed electrode polarity reversal experiments, as shown in Figure S25 (Supporting Information), to prove the legitimacy of the device's signal output. Furthermore, the device's maximum power density output of 27 mW m^−^^2^ was attained with a load resistance of 1 MΩ as illustrated in Figure S23g (Supporting Information). Finally, a cyclic stability test was performed on the stress sensor, as illustrated in Figure S23h (Supporting Information). Under a high‐frequency motion at 10 Hz, the C_6_H_5_N(CH_3_)_3_CdBr_2_Cl_0.75_I_0.25_‐based stress sensor exhibited good output stability, up to 100 000 cycles.

### 2.5 Practical Application of OIHPF‐Based Energy Harvesters and their Potential for Large‐Scale Manufacturing

**Figure 6:**
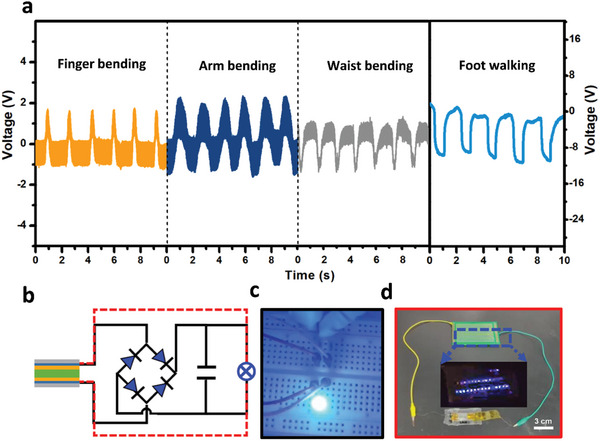
Practical application display of OIHPF‐based PEH: a) TMCM‐CdBrCl_2_‐based PEH used to monitor human body gestures; b) Circuit diagram w/wo the full‐wave four diode bridge rectifier; c) TMCM‐CdBrCl_2_‐based PEH with full‐wave four diode bridge rectifier to light on a commercial blue LED (black circuit); d) C_6_H_5_N(CH_3_)_3_CdBr_2_Cl_0.75_I_0.25_‐based PEH directly connected to light 27 commercial LEDs under the repetitive hand tapping without extra storage system (red circuit).

The practical energy harvesting applications of OIHPFs and their potential for large‐scale manufacturing are displayed in **Figure** **6**. For self‐powered wearables, biomechanical motions are regarded as ecologically beneficial energy sources. As seen in Figure 6a–d, the TMCM‐CdBrCl_2_ device was fixed to several parts of the human body in order to evaluate its availability and viability for piezoelectric energy harvesting (PEH). During the periodic contraction and relaxation stages, the device displays stable signals, indicating that it has a high capacity to harvest energy from human body movement (Figure 6a). With extra help of rectifying and storing elements, as seen in Figure 6b and Movie S3 (Supporting Information), the PEH operating under a bending frequency of 3 Hz was utilized to light a blue light‐emitting diode (LED, Figure 6c). The outcome shows that TMCM‐CdBrCl_2_ film has the capacity to produce sufficient energy for consumer electronics in real‐world applications.

In contrast to the TMCM‐CdBrCl_2_‐based PEH, we made major changes to demonstrate the energy harvesting capability of C_6_H_5_N(CH_3_)_3_CdBr_2_Cl_0.75_I_0.25_ film. As shown in Figure 6d and Movie S4 (Supporting Information), without the aid of any rectifying or storing components, it successfully lights up 27 commercially available blue LEDs by repeatedly tapping the device with a palm, thoroughly proving the device's outstanding piezoelectric output capabilities.

More importantly, we successfully obtained C_6_H_5_N(CH_3_)_3_CdBr_2_Cl_0.75_I_0.25_ thin films on an A4‐sized ITO/PET substrate using the smallest available 34G metal needle. As played in Movie S5 (Supporting Information), a large‐area printing of C_6_H_5_N(CH_3_)_3_CdBr_2_Cl_0.75_I_0.25_ film was achieved at *H* = 6 mm, U = 5000 V, *d* = 5 mm, and *v* = 8 mm s^−1^. This truly shows the viability of EHD‐spray printing for large‐area preparation of OIHPF thin films, creating a critical research basis for future larger nozzle printing, multi‐nozzle efficient printing, and even roll‐to‐roll mass manufacturing of piezoelectric films. We believe that this technique is not restricted to the large‐scale fabrication of piezoelectric films, but also provides a key research strategy for the multifunctional application of other ionic crystals.

### 2.6 Other Environmental Considerations

This study introduces a pioneering strategy employing EHD printing for the rapid and efficient fabrication of large‐area OIHPF crystals and films. However, their environmental stability, recyclability, and potential environmental impact are critical concerns in practical applications, due to the inherent weakness of hybrid perovskites, such as their sensitivity to moisture, heat, and light.^[^ ^36^ ^]^

1. Environmental Stability: To evaluate the environmental stability of EHD‐sprayed TMCM‐CdBrCl_2_, the micro‐ferroelectric property and macro‐piezoelectric output performance of long‐term RT stored TMCM‐CdBrCl_2_ film (vacuum, over one year) and film‐based PENG (air, over half an year) were explored, respectively. As shown in Figure S26 (Supporting Information), the granular and short rod‐like interwoven morphology of TMCM‐CdBrCl_2_ results from the temperature‐induced re‐growth as mentioned earlier. Notably, the characteristic semi‐domains and well‐defined domain walls of TMCM‐CdBrCl_2_ were consistent with earlier PFM findings (as shown in Figure 3a–e), confirming its robust environmental stability at the microscopic scale. Moreover, performance evaluations of the TMCM‐CdBrCl_2_‐based PENG, presented in Figure S27 (Supporting Information), demonstrated that the peak output voltage of the air‐exposed thin‐film device declined by merely 5% compared to its initial value under identical testing conditions (4.64%, 1 Hz). After subjecting the device to ultrasonic treatment at 40 kHz for 40 min in water, its output performance exhibited a reduction of 14.65% compared to the device that was not immersed. Given the rough packaging of the device with PI tape instead of PDMS, known for its superior barrier properties,^[^ ^37^ ^]^ this result is deemed acceptable. Figure S28 (Supporting Information) shows the FTIR spectrum of TMCM‐CdBrCl_2_ films (air, three days), without detecting any ‐OH characteristic peaks in 3200–3600 cm^−1^. This absence of peaks indicates that neither bonded nor free water is present in the film, thereby demonstrating its excellent short‐term moisture resistance. Additionally, we also performed PXRD measurements on the prepared TMCM‐CdBrCl_2_ film (vacuum, 10 months) at different time. As shown in Figure S29 (Supporting Information), neither the peak positions nor the peak intensities changed or decreased, indicating the remarkable environmental resilience of the prepared film. Furthermore, as shown in Figure S30 (Supporting Information), the precursor solutions of TMCM‐CdBrCl_2_ and C_6_H_5_N(CH_3_)_3_CdBr_2_Cl_0.75_I_0.25_, after being exposed to air for one and a half years, exhibited no impurities, precipitation, or discoloration, further confirming their good environmental stability. This study seeks to validate the strong feasibility and wide‐ranging applicability of EHD printing in fabricating large‐scale OIHPF films, with a primary focus on 1D OIHPFs, such as TMCM‐CdBrCl_2_ and C_6_H_5_N(CH_3_)_3_CdBr_2_Cl_0.75_I_0.25_, owing to their exceptional piezoelectric performance.^[^ ^6^, ^20^ ^]^ Furthermore, 1D OIHPFs demonstrate markedly enhanced environmental stability compared to their higher‐dimensional analogs.^[^ ^38^ ^]^ Nonetheless, it is strongly advised that EHD printing, film sintering, and device packaging for others OIHPFs‐especially the sensitive 3D Pb‐based perovskite solar cell materials,^[^ ^39^ ^]^ be performed in a controlled environment, such as a glove box, to ensure long‐term stability.
2. Sustainability: Regarding the sustainability of EHD‐printed OIHPFs, it is not a significant challenge due to the excellent solution processability of OIHPF materials and the inherent technical advantage of EHD printing. Similar to conventional thin‐film fabrication methods, such as spin coating, EHD printing utilizes ***EF***‐assisted solvent evaporation to induce solute crystallization. Notably, EHD printing is classified as an additive manufacturing technique with minimal material loss, thus providing substantial benefits in material conservation and raw material efficiency. For recycling, OIHPFs can be readily concentrated via multilayer printing in designated regions, subsequently collected, and dried for reuse. The measurements of d_33_, temperature‐dependent dielectric analysis (Figure S6, Supporting Information), and electric hysteresis loop (Figure S17, Supporting Information) on TMCM‐CdBrCl_2_ polycrystalline pellet were all carried out through this strategy, proving the feasibility of EHD‐printed materials for sustainability development.
3. Environmental impact: The environmental impact of EHD‐printed OIHPFs is intrinsically tied to the safety of their composition, which can be mitigated through the outstanding compositional tunability and solution processability of these materials.^[^ ^40^ ^]^ Consequently, Pb‐free OIHPFs emerge as promising candidates for the next generation of flexible devices. TMCM‐CdBrCl_2_, as one of the Pb‐free OIHPFs, demonstrates potential for piezoelectric applications. For long‐term safety considerations, however, metal‐free molecular perovskite ferroelectrics, such as MDABCO‐NH_4_I_3_,^[^ ^41^ ^]^ are strongly recommended, remaining compatible with EHD printing from a material's perspective.

## 3 Conclusion

In conclusion, we propose a facile one‐step strategy of constructing large‐scale and poling‐free OIHPF piezoelectric crystals or thin films based on the principle of ***EF***‐induced in‐situ crystallization. With varying printing conditions, the TMCM‐CdBrCl_2_ building blocks present tunable morphology and sizes, generating TMCM‐CdBrCl_2_ films with tailored density and crystallinity. Of particular significance is that the optimal TMCM‐CdBrCl_2_ film exhibits much enhanced piezoelectric coefficient of 5.8 times compared with reference sample. Meanwhile, it also shows high signal stability over 200 000 bending tests. The technique is also useful for producing other kinds of OIHPFs crystals and films, offering a generally applicable strategy for constructing high‐performance large‐scale OIHPF films for flexible electronic devices.

## 4 Experimental Section

### Preparation of OIHPF Precursor Ink

The TMCM‐CdBrCl_2_ precursor ink was prepared by mixed Trimethylchlorome‐thylammonium bromide (TMCM‐Br) with equimolar amounts of CdCl_2_ in an H_2_O/DMF solvent mixture at a volume ratio of 3:2. The mass concentration of the ink was 100 mg mL^−1^. The obtained precursor solution was ultrasonicated for 30 min and then filtered by a 0.22 µm nylon filter for further removal of the residual agglomerates.

The C_6_H_5_N(CH_3_)_3_CdBr_2_Cl_0.75_I_0.25_ precursor ink was created by dissolving a mixture of C_6_H_5_N(CH_3_)_3_‐Cl, C_6_H_5_N(CH_3_)_3_‐I, and CdBr_2_∙4H_2_O in stoichiometric proportions within the same H_2_O/DMF solvent system. Subsequently, a precursor ink with a mass concentration of 88 mg mL^−1^ was obtained following the same preparation protocol.

### Deposition of OIHPF Crystals or Films Through EHD Printing, Spin‐Coating or Drop‐Coating

Detailed information about the EHD printer utilized in this study, as illustrated in Figure S1 (Supporting Information), is provided in Text S1 (Supporting Information). ITO‐coated PET or glass, and silicon wafers with thicknesses of 150 nm, 125 µm, 1 mm, and 1 mm were used as printing substrates. Before use, the substrates undergo hydrophilic treatment using oxygen plasma (100 W, 200 sccm, 5 min) to improve surface wettability and promote uniform ink deposition. At ambient temperature, the TMCM‐CdBrCl_2_ or C_6_H_5_N(CH_3_)_3_CdBr_2_Cl_0.75_I_0.25_ precursor ink was injected into a 1 mL plastic syringe. During the EHD printing process, the applied ***EF*** was gradually increased until it overcame the surface tension of precursor solution, enabling the formation of thin jets or numerous micro‐nanodroplets. OIHPF crystals of various shapes and sizes, or films, were deposited onto the selected substrate by adjusting parameters, such as ***H***, ***U***, ***P***, ***d***, ***v***, ***D***, and printing layers. Post‐deposition, all samples were vacuum‐dried overnight at controlled ***T_S_*** to ensure complete solvent removal and to optimize the film crystallinity.

The spin‐coated TMCM‐CdBrCl_2_ films were fabricated at varying speeds (2500–4000 rpm, 30 s) using 30 µL of precursor solution with concentrations ranging from 100 to 300 mg mL^−1^, followed by sintering in a vacuum oven at 75 °C overnight. The optimal film, prepared at 3500 rpm, exhibited a thickness of 1.5 µm.

For the drop‐coated TMCM‐CdBrCl_2_ film, 30 µL of precursor solution was directly dispensed onto the substrate, dried at room temperature for 30 min, and subsequently placed in a vacuum oven at 75 °C for overnight drying.

### Preparation of Piezoelectric Sensors or Energy Harvesters

A 10:1 weight ratio of PDMS resin and curing agent (Sylgard 184, Dow Corning, USA) was spin‐coated at 3000 rpm for 45 s onto the surface of EHD‐sprayed or spin‐coated TMCM‐CdBrCl_2_ or C_6_H_5_N(CH_3_)_3_CdBr_2_Cl_0.75_I_0.25_ film deposited on ITO/PET substrates, followed by partial curing at 83 °C for 2 min. Another OIHPF‐deposited ITO/PET substrate was subsequently pressed onto the PDMS layer (thickness: 758 µm), which was then dried at 83 °C for an additional 10 min. The total thickness of the device is ≈1 mm. The finalized OIHPF‐based PENG devices were fabricated by attaching ITO electrodes and copper wires with silver gel, followed by lamination with Kapton tape to ensure electrical isolation. The as‐fabricated device featured a total active area of 1.5 cm^2^.

The PDMS reference sample was prepared by spin‐coating PDMS directly onto bare ITO/PET substrates, maintaining consistency with all other fabrication procedures.

It is worth noting that none of the film‐based PENGs in this investigation went through any extra high‐voltage poling.

### Characterization Methods

The surface morphology of the as‐synthesized OIHPF films or crystals was examined using a scanning electron microscope (SEM, S‐4800, Hitachi, Japan), and an optical microscope (MJ30, Micro‐shot Technology Co., Ltd., Guangzhou). Additionally, the elemental distribution of samples was analyzed through energy‐dispersive X‐ray spectroscopy (EDS). The film coverage was quantitatively evaluated using ImageJ software. Raman and second‐harmonic generation (SHG) spectroscopy were conducted using a Metatest ScanPro Laser Scanning System (ScanPro Advance, Metatest), featuring a Nd‐YAG laser with a wavelength of 1064 nm. Atomic force microscopy (AFM) and piezoresponse force microscopy (PFM) measurements were carried out using a commercial piezoresponse microscope (Cypher S, Oxford Instruments) to evaluate the thickness, domain structure, switching behavior, and local piezoresponse of the thin films. The scanning angle was fixed at 90° during the measurements. Fourier transform infrared (FTIR) spectroscopy was conducted using a Bruker INVENIO‐R spectrometer (Germany) over a wavenumber range of 400–4000 cm⁻¹. Polarization‐electric field (P–E) hysteresis loops were recorded using a TF Analyzer 3000 (aixACCT, Germany) operating in leakage compensation mode and equipped with a Sawyer‐Tower system. The measurements were performed on a compressed TMCM‐CdBrCl_2_ polycrystalline pellet with dimensions of 3 mm in thickness and 5 mm in diameter at room temperature. The pellet was fabricated by hot‐pressing the multi‐layer EHD‐sprayed sample at 75 °C overnight, followed by coating both sides with silver glue. Differential scanning calorimetry (DSC) measurements were performed using a DZ‐DSC300C instrument (Nanjing Dazhan Instrument, China) by heating and cooling the EHD‐sprayed sample at a rate of 5 K min^−1^ in an aluminum crucible under a nitrogen atmosphere. The thermal stability of TMCM‐CdBrCl_2_ was evaluated by a Thermomechanical Analyzer (Mettler TMA/SDTA^2+^) at a heating rate of 10 K min^−1^ in aluminum crucibles under same atmosphere. Dielectric measurements for the TMCM‐CdBrCl_2_ pellet were carried out using an E4980A LCR meter (Keysight, USA), equipped with a heated plate. Powder X‐ray diffraction (PXRD) measurements were performed using a MiniFlex 600 diffractometer (Rigaku, Japan) with a Cu Kα radiation source (λ = 1.5406 Å, 40 kV, 15 mA) over a 2θ range of 5°–50°. The obtained data were further analyzed using the “curve‐fitting” function in Origin 8.5 to quantify variations in crystallinity. The analysis followed several key steps: 1) Baseline subtraction: Background noise was removed using the Peak Analyzer tool in Origin 8.5 (Analysis > Peaks and Baseline > Create Baseline), ensuring a more accurate data representation; 2) Identification of crystalline peaks: Individual crystalline peaks were identified, marked, and their areas calculated; 3) Identification of overall peak area: A single broad peak encompassing all crystalline and amorphous peaks was fitted to determine the total peak area; 4) Calculation of crystallinity using the formula:

(8) $$\mathrm{Crystallinity}(\%)=\frac{Areaofcrystallinepeaks}{Areaofallpeaks\left(crystalline+amorphous\right)}\times 100$$

The piezoelectric performance of the films was assessed using a Keithley 6514 electrometer (USA), coupled with either a linear motor (R‐LP4, Nano Energy, Beijing) or a universal testing machine (FlexTest‐F‐C, Hunan Nano‐Up Electronics Technology Co., Ltd) in pushing mode to conduct vertical compression and bending tests, respectively. The direct piezoelectricity of polycrystalline TMCM‐CdBrCl_2_ pellet was assessed using a ZJ‐4AN type quasi‐static d_33_ analyzer (Chinese Academy of Sciences, Beijing). Before measurement, the pellet was polarized in air using an electrical poling field of 3.3 for 3 h, with a HV‐153P2 high voltage DC power supply (Tianjin Shenghuo, China).

## Conflict of Interest

The authors declare no conflict of interest.

## Supporting information



Supporting Information

Supplemental Movie 1

Supplemental Movie 2

Supplemental Movie 3

Supplemental Movie 4

Supplemental Movie 5

## Data Availability

The data that support the findings of this study are available from the corresponding author upon reasonable request.
