# Supplementary material for: Facile Fabrication of Large‐Area and High‐Quality Organic–Inorganic Hybrid Perovskite Ferroelectric Films Through Electrohydrodynamic Printing
Source: Adv Sci (Weinh). 2025 Apr 1;12(21):2414122. doi: 10.1002/advs.202414122 (PMC12140340; doi:10.1002/advs.202414122)
Supplement: Supplementary file 1 — Supporting Information [file ADVS-12-2414122-s004.docx]

**Facile Fabrication of Large-Area and High-Quality Organic-Inorganic Hybrid Perovskite Ferroelectric Films through Electrohydrodynamic Printing**

Jingjing Luo^1, #^, Zhongqi Ren^1, #^, Xue Qi^1^, Qiang Pan^2^, Dong Li^1^, Yuan Xiong^2^, Jie Yao^2^, Haipeng Liu^1^,

Suzhu Yu^1, *^, Jun Wei^1,^ ^*^

1. Shenzhen Key Laboratory of Flexible Printed Electronics Technology, School of Materials Science and Engineering, Harbin Institute of Technology, Shenzhen, 518055, China

2. Jiangsu Key Laboratory for Science and Applications of Molecular Ferroelectrics, Southeast University, Nanjing, 211189, People’s Republic of China

# The two authors contributed equally to this work as first authors

Corresponding authors e-mail:

[szyu@hit.edu.cn](mailto:szyu@hit.edu.cn); [junwei@hit.edu.cn](mailto:junwei@hit.edu.cn)

The PDF file includes:

Supplementary Text S1 to S4

Figs. S1 to S30

Table S1

Other Supplementary Material for this manuscript includes the following:

Movie S1

Movie S2

Movie S3

Movie S4

Movie S5

**Supplementary Text S1:**

As depicted in Fig. S1, the EHD printer comprises several integral components working in unison to ensure the precise deposition of OIHPF-based ink onto the substrate. The system includes a 1 mL plastic syringe equipped with interchangeable glass or metal nozzles of varying inner diameters, connected to an air compressor (OTS-980, Outstanding Industry & Trade Co., Ltd., Taizhou) for air pressure generation. The setup also includes a high-voltage power supply (HV-153P2, Torch Technology Co., Ltd., Tianjin) and a grounded X-Y translation stage that accommodates various printing substrates. Additionally, the system features a Z-axis lifting platform and a sophisticated control mechanism comprising cameras and a light source integrated into a PC terminal to enable real-time monitoring and precise adjustment of nozzle conditions.


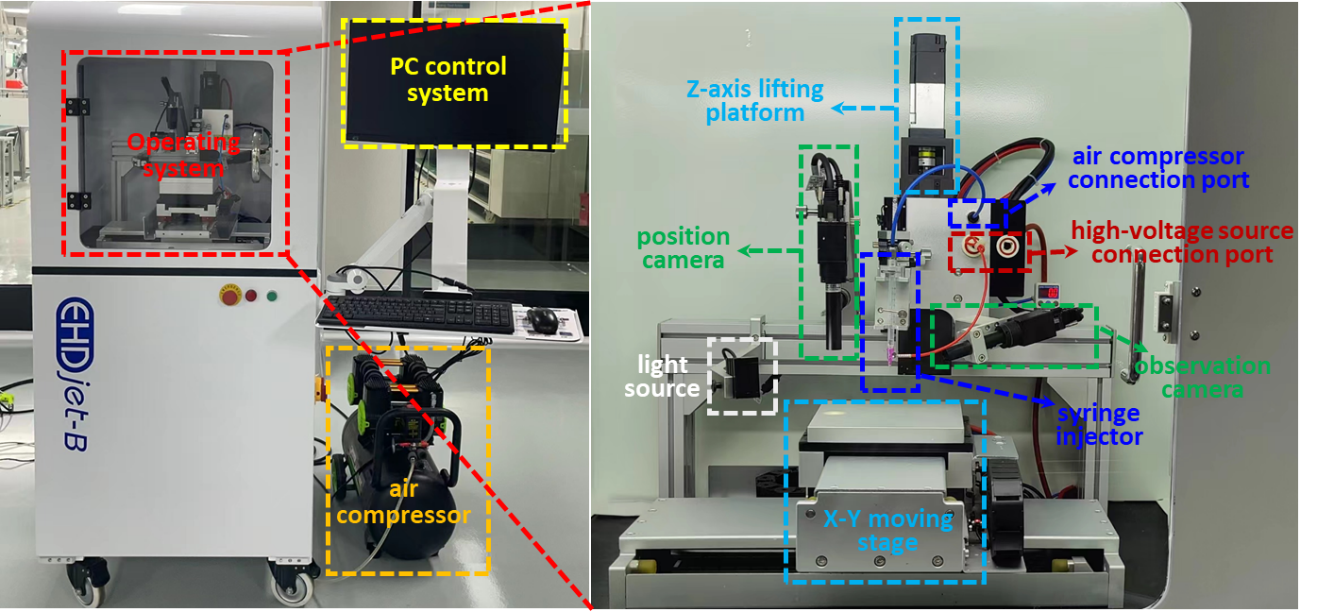


**Fig. S1 The picture of the EHD printer.**

**Supplementary Text S2:**

The printing path in this work uses a serpentine "S" routing mode, with the printing width ***L*** chosen arbitrarily and the line distance ***d*** set based on the actual printing requirements.


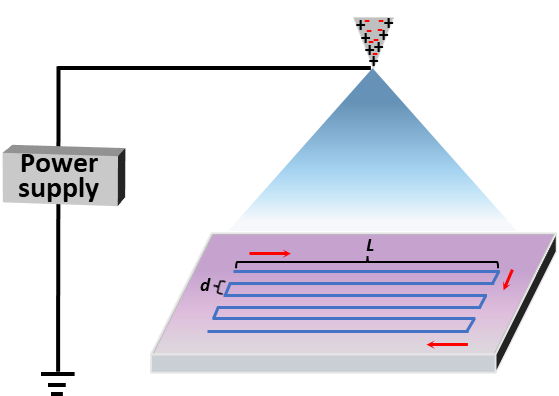


**Fig. S2 Ion distribution near the nozzle tip.**

**Supplementary Text S3:**

For safety, we began with a low ***U*** of 2000 V and gradually decreased ***H*** until ink began to spurt out. This indicated that the electric field force was just enough to overcome the surface tension of the TMCM-CdBrCl_2_ precursor solution, establishing 9 mm and 2000 V as the upper limit for ***H*** and the lower limit for ***U***, respectively. Further decreasing ***H*** at 2000 V showed that a regular film could not be obtained below 3 mm, defining 3 mm as the lower distance limit.

Next, we fixed ***H*** at 3 mm and gradually increased ***U*** to determine the upper voltage limit. When ***U*** exceeded 6000 V, electric sparks appeared, and the nozzle became blocked due to excessive charge concentration and rapid solvent evaporation. Therefore, the working window made up of ***H*** and ***U*** was set to 3-9 mm and 2000-6000 V, respectively.


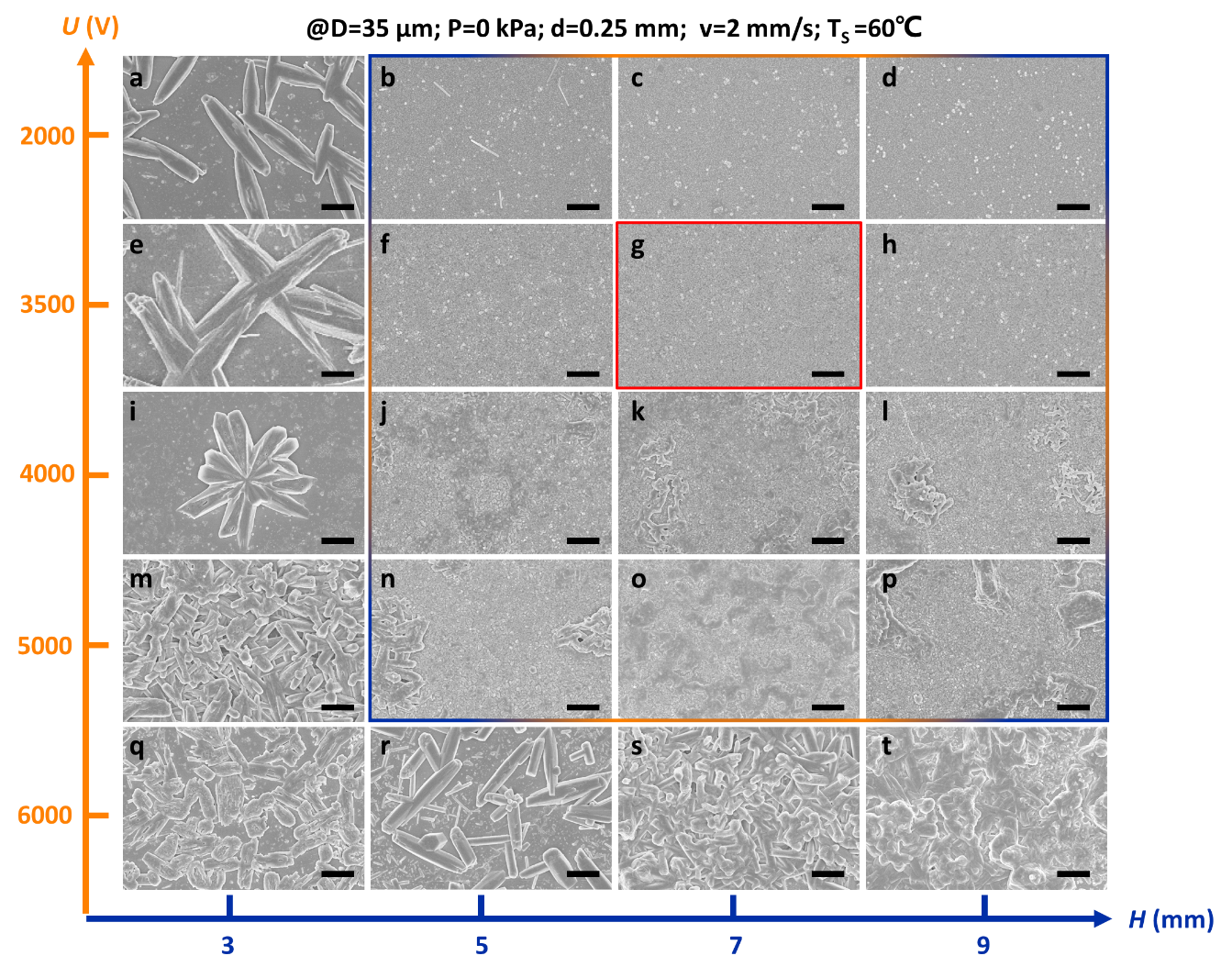


**Fig. S3 SEM images of TMCM-CdBrCl_2_ films and crystals fabricated using the “Far-field” spraying mode (indicated by the interwoven blue and yellow lines) and the “Near-field” jetting mode (denoted by the remaining samples). The scale bar is 3 μm.**

**Supplementary Text S4:**

**The morphological evolution of TMCM-CdBrCl_2_ under different printing modes**

1. **Under the “Near-field” jetting mode**

When examining the “Near-field” jetting mode with ***H*** set at 3 mm, it is evident that as ***U*** increases, the morphology of TMCM-CdBrCl_2_ undergoes distinct transformations. Initially, the material exhibits an independent rod-like structure (Fig. S3a). As ***U*** continues to rise, two spindle-shaped structures intertwine to form a cross-like configuration (Fig. S3e), which eventually aggregates into a flower-like form (Fig. S3i). This phenomenon occurs below 4000 V and can be explained by the rising surface-area-to-volume ratio and its associated higher ***ΔG_S_***. Consequently, the crystals aggregate to reduce their surface area, thereby lowering the surface energy and making the system more stable. However, when ***U*** continues to increase, the flower-like crystal structure tends to break into irregular short sticks (Figs. S3 m and q), indicating that excessive charge concentration causes crystal structure degradation. This degradation can be alleviated, as evidenced by the repeated appearance of the rod-like structure when ***EF*** is reduced through increasing ***H*** (Fig. S3r). With a further rise in ***H***, the rod-like structure is replaced by particles due to the weakened ***EF*** force (Figs. S3 s and t).

1. **Under the “Far-field” spraying mode**

In the “Far-field” spraying mode, it is evident that an increase in ***U*** at a fixed ***H*** displays similar trends where larger particles form denser films, but beyond a threshold of 3500 V, their homogeneity begins to deteriorate. Additionally, the films formed at higher ***H*** with fixed ***U*** exhibit worse surface states after a threshold of 7 mm. These issues can be attributed to the printing instability induced by inappropriate ***EF*** forces resulting from excessively large ***U*** or ***H***.


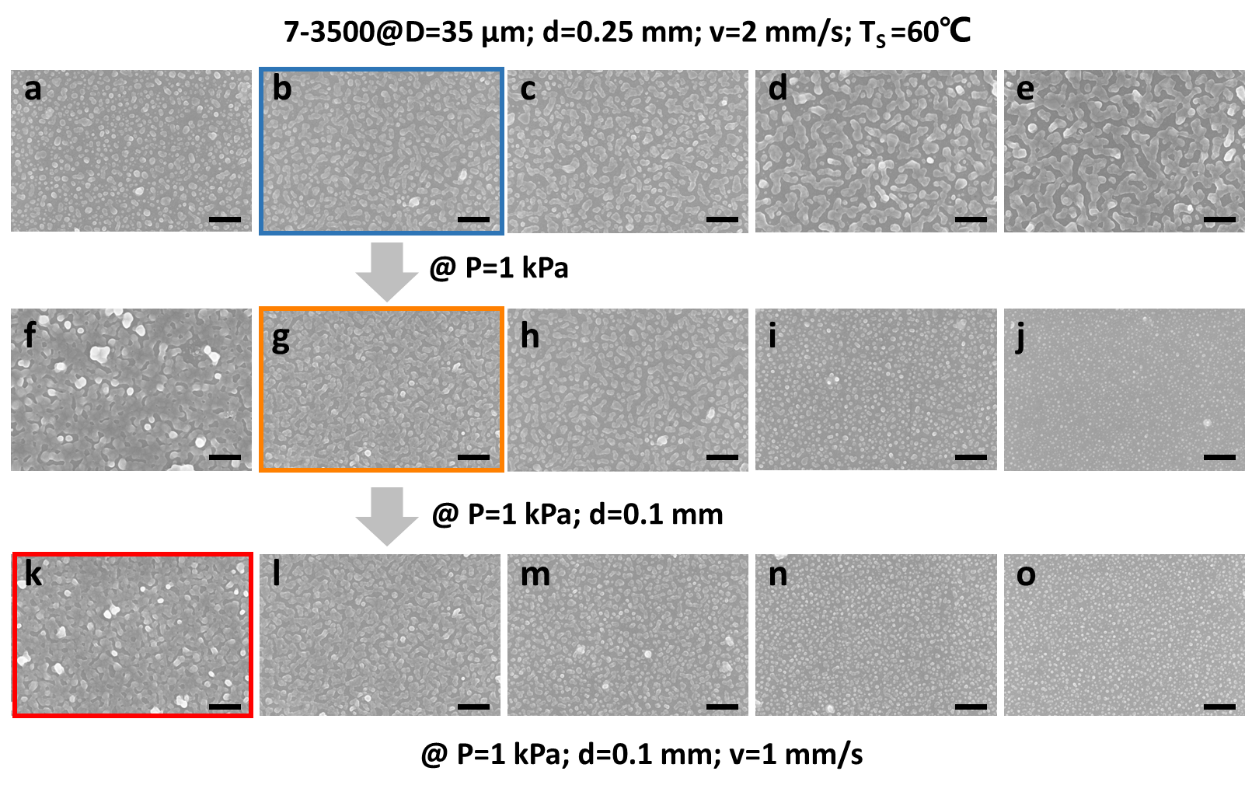


**Fig. S4 SEM images showing the optimization process of** **printed TMCM-CdBrCl_2_ films through controlling *P*, *d* and *v***: **a-e).** ***P*** set as 0, 1, 5, 10, and 15 kPa, respectively; **f-j)**. ***d*** controlled as 0.05, 0.1, 0.25, 0.5, and 1 mm, respectively; **k-o).** ***v*** specified as 1, 2, 4, 6, and 8 mm/s, respectively (scale bar = 500 nm).


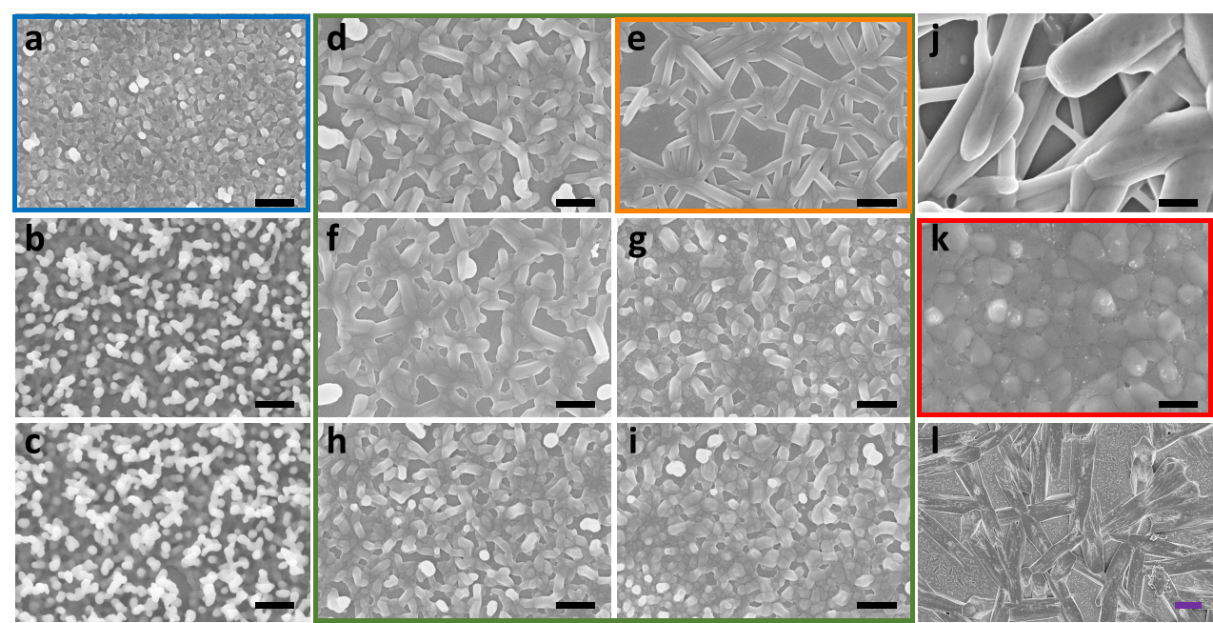


**Fig. S5 SEM images of TMCM-CdBrCl_2_ films printed under different conditions**: **a-c)** Printing layers of 1, 3, and 5 at 60℃; **d-i)** Increasing ***T_S_*** of 70, 75, 80, 85, 90, and 120℃; and **j-l)** Varying ***D*** of 15, 25, and 45 μm, respectively (black scale bar = 500 nm, purple scale bar = 10 μm).

**
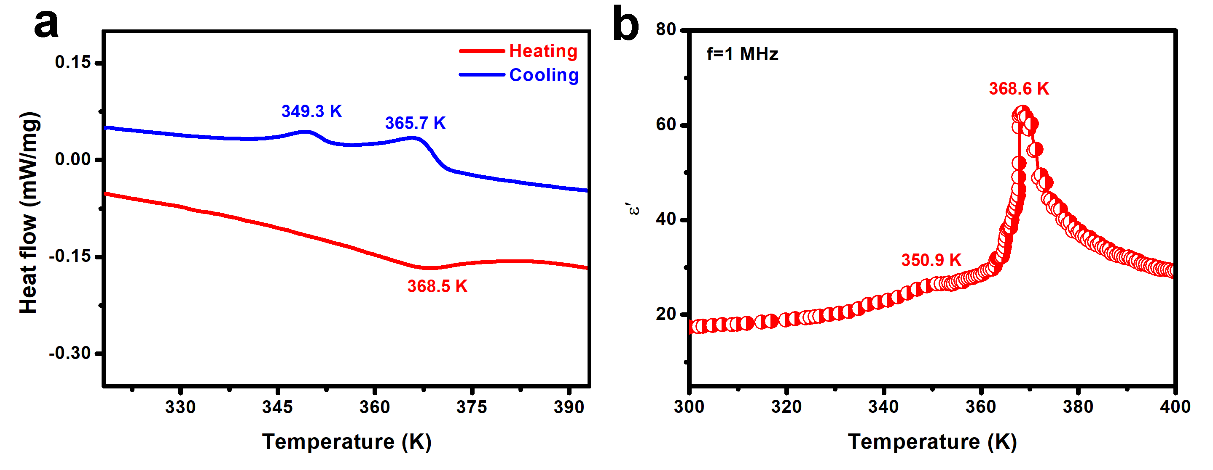
**

**Fig. S6 DSC a) and dielectric b) measurements of TMCM-CdBrCl_2_.**

**

**

**Fig. S7 Thermomechanical analysis of TMCM-CdBrCl_2_.**

**
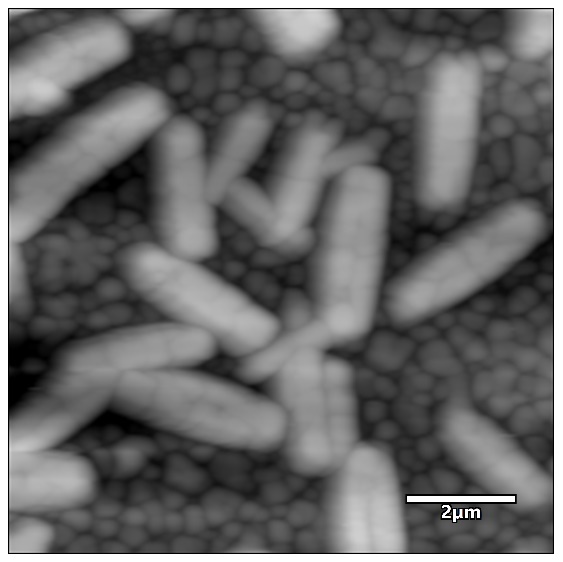
**

**Fig. S8 AFM image of TMCM-CdBrCl_2_ film with a short sintering time of 6 h.**


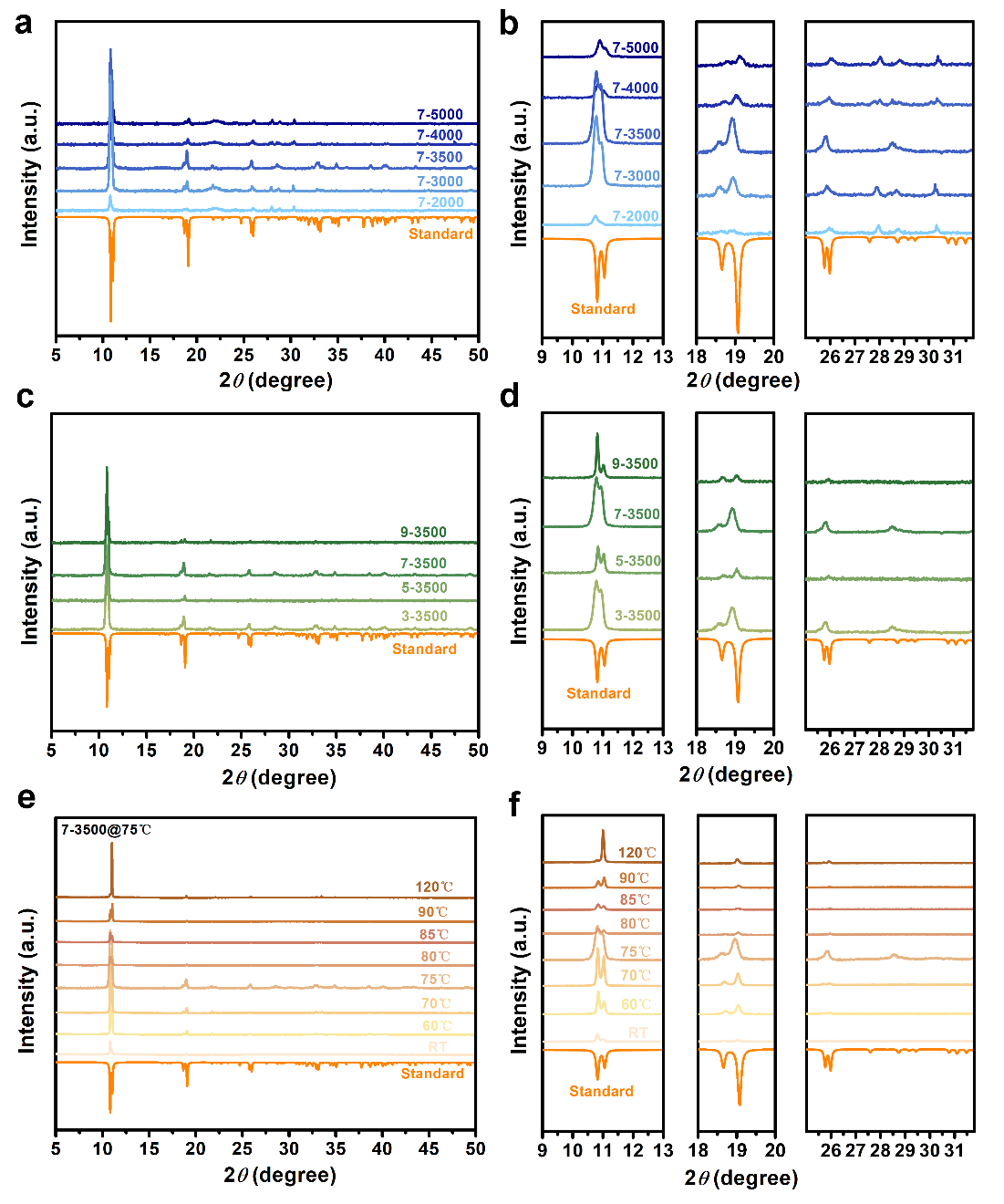


**Fig. S9 PXRD patterns of prepared TMCM-CdBrCl_2_ films with varying a, b) *U*, c, d) *H***, and **e, f) *T_S_***.

**
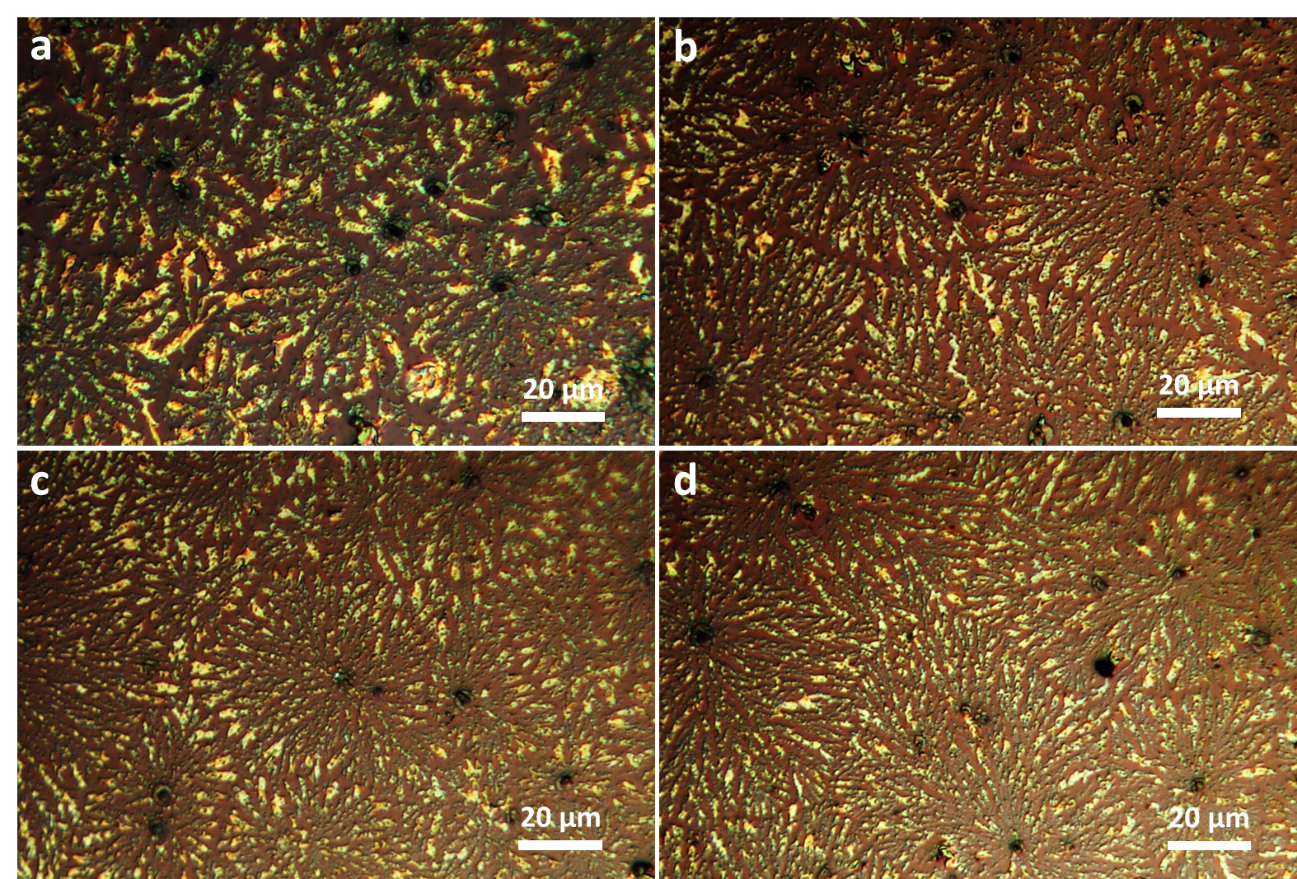
**

**Fig. S10 Optical Microscopy Images of spin-coated TMCM-CdBrCl_2_ films prepared at a)** 2500 rpm, **b)** 3000 rpm, **c)** 3500 rpm, and **d)** 4000 rpm, respectively.

**
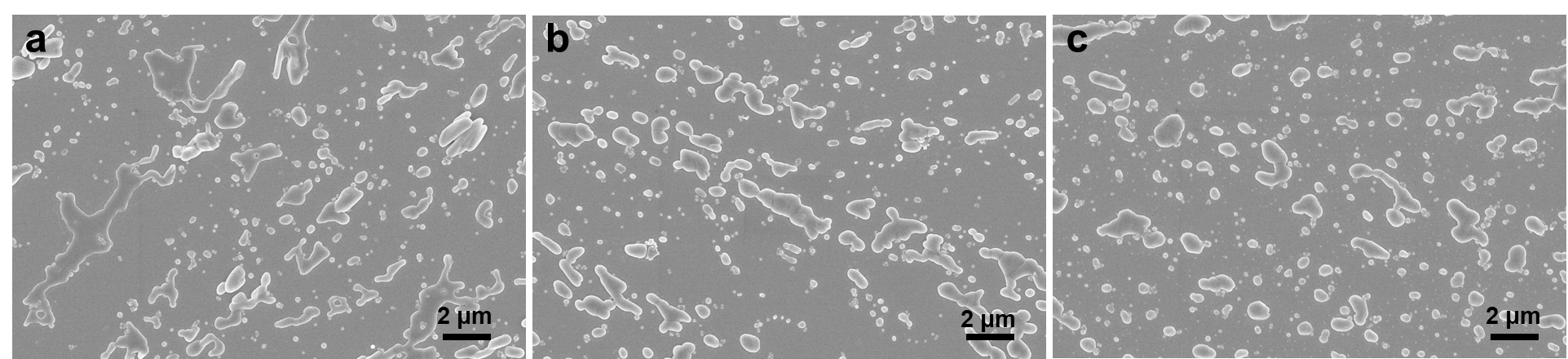
**

**Fig. S11 SEM images of spin-coated TMCM-CdBrCl_2_ films prepared at 3500 rpm in different precursor solution concentrations**: **a)** 100 mg/mL; **b)** 200 mg/mL; and **c)** 300 mg/mL, respectively.

**

**

**Fig. S12 PXRD result of TMCM-CdBrCl_2_ film in comparison with the standard peak**[^1^](#_ENREF_1)**.**

**

**

**Fig. S13 The Rietveld-refined PXRD result of the TMCM-CdBrCl_2_ film.**


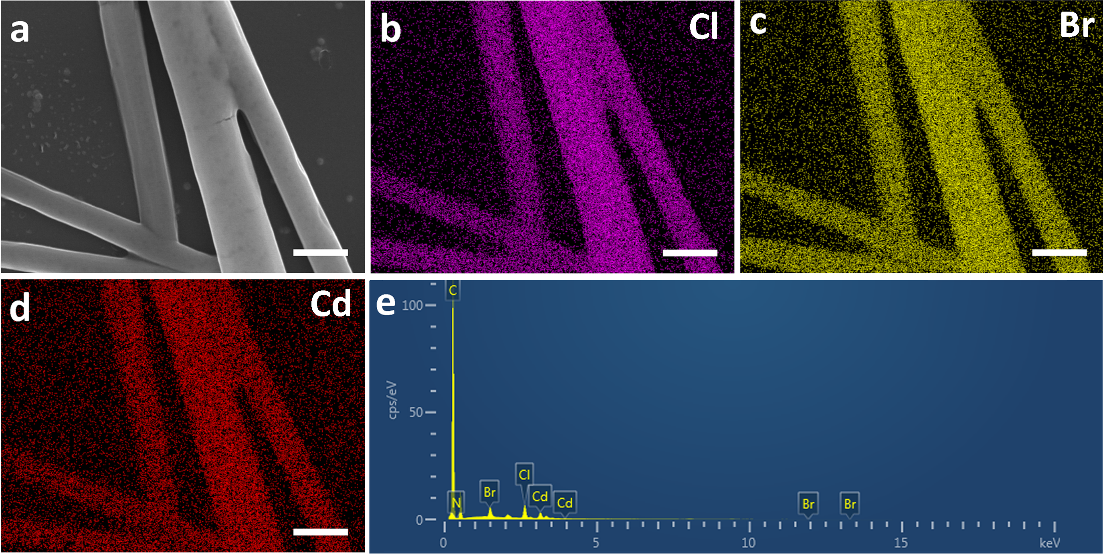


**Fig. S14 EDS mapping results of the rod-like TMCM-CdBrCl_2_ crystal (scale bar = 1 μm).**


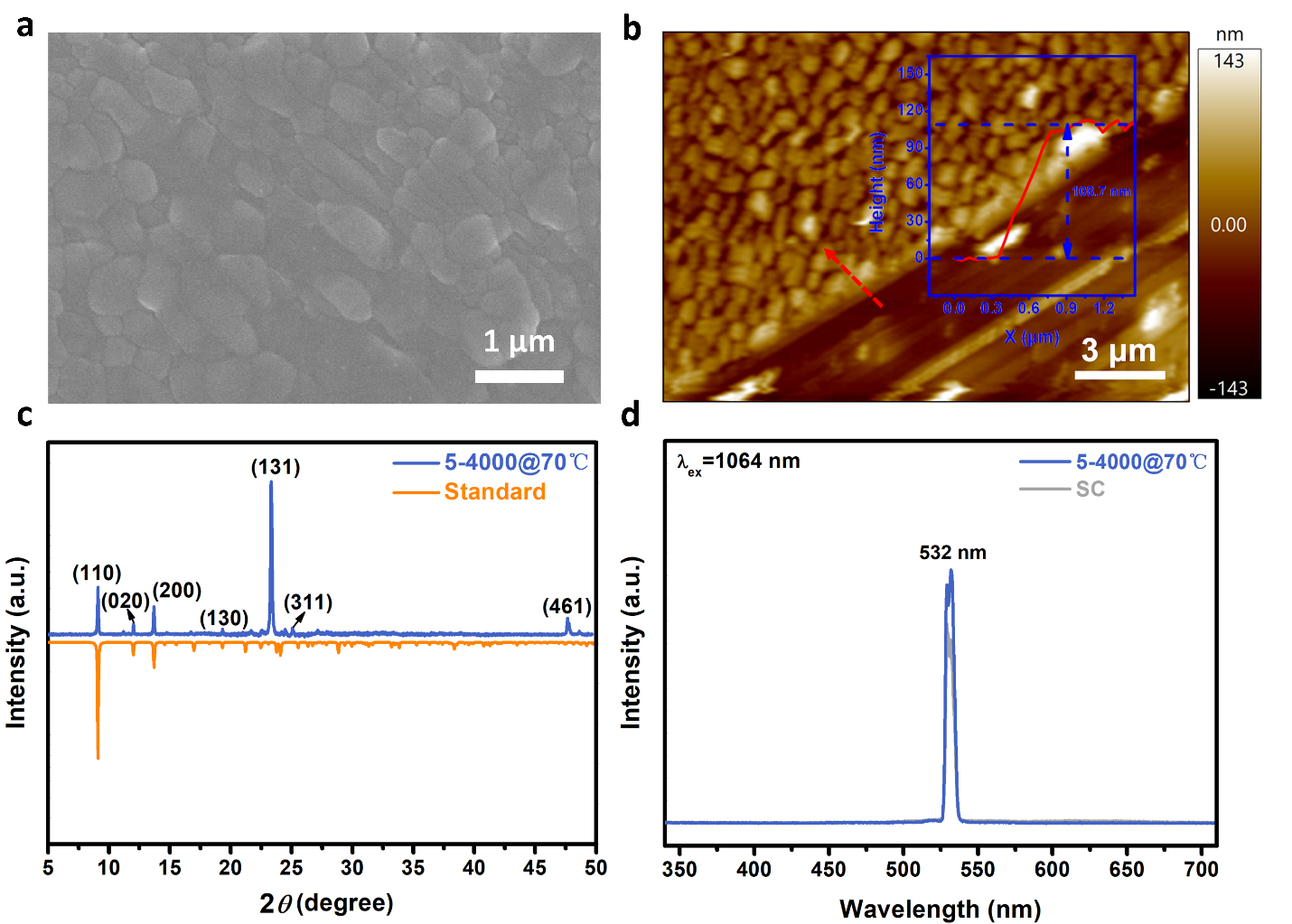


**Fig. S15 EHD-sprayed single-layer C_6_H_5_N(CH_3_)_3_CdBr_2_Cl_0.75_I_0.25_ film prepared at 5-4000@70℃, v= 1 mm/s, P= 1 kPa, and d= 0.25 mm**: **a)** SEM image; **b)** AFM image with an insert of thickness result; **c)** PXRD pattern compared with the standard one; and **d)** SHG spectra results.


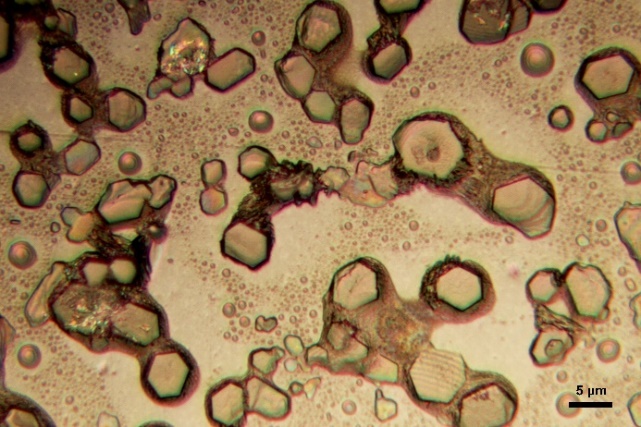


**Fig. S16 “Near-field” jetting mode used to produce C_6_H_5_N(CH_3_)_3_CdBr_2_Cl_0.75_I_0.25_ crystals on ITO glass.**

**

**

**Fig. S17 P–E hysteresis loop of TMCM-CdBrCl_2_ compacted pellet.**

**
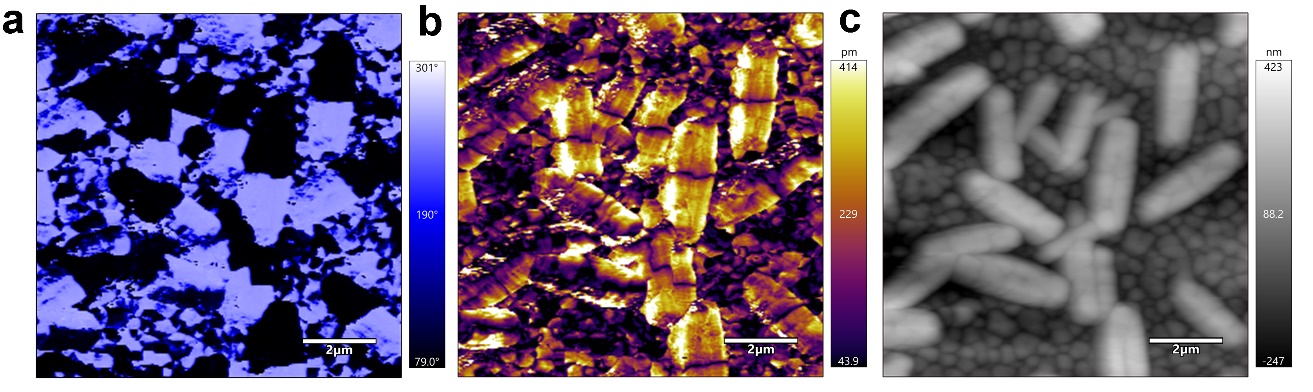
**

**Fig. S18 PFM images of TMCM-CdBrCl_2_ film with a sintering time of 6 h**: **a)** Lateral phase image; **b)** Lateral amplitude image; and **c)** Topographic image.

**
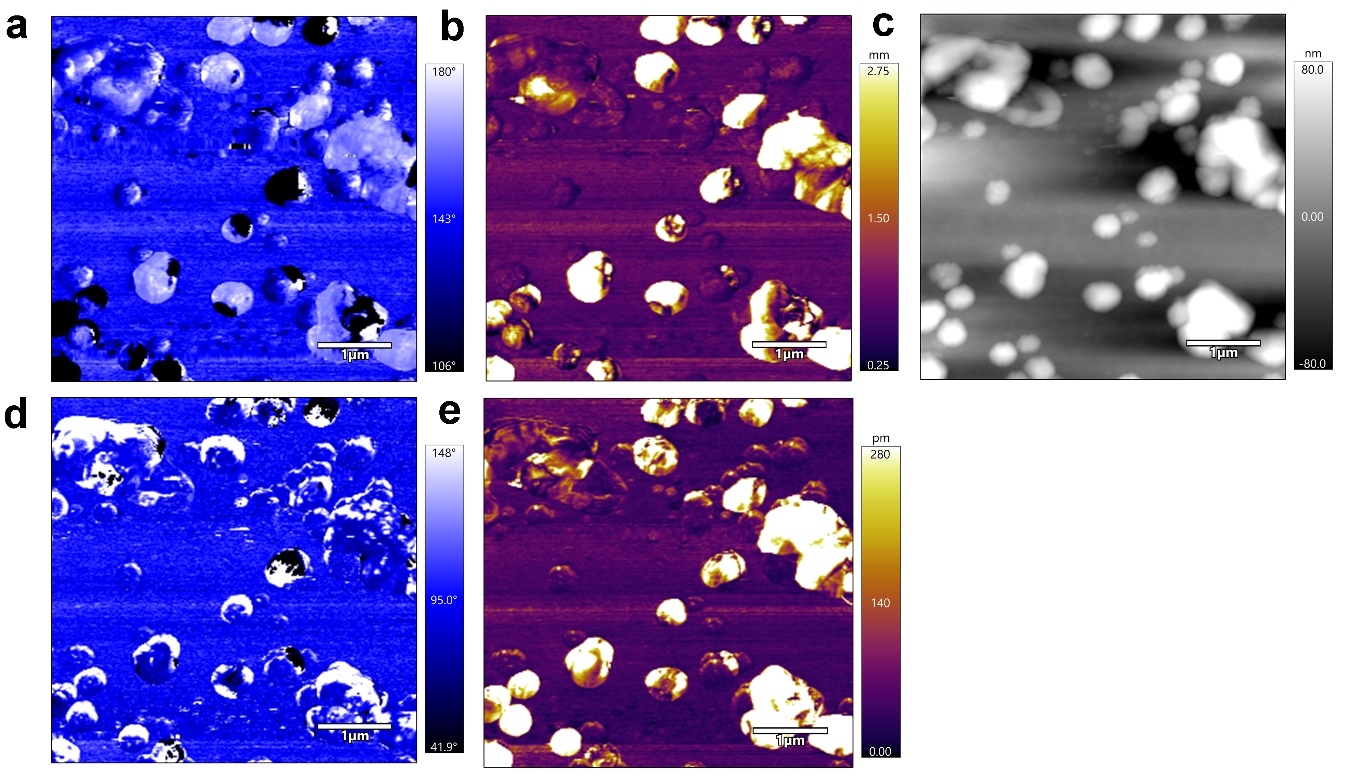
**

**Fig. S19 PFM images of spin-coated TMCM-CdBrCl_2_ film**: **a)** and **b)** Lateral PFM phase and amplitude images, respectively; **c)** Topographic image; **d)** and **e)** Vertical PFM phase and amplitude images, respectively.


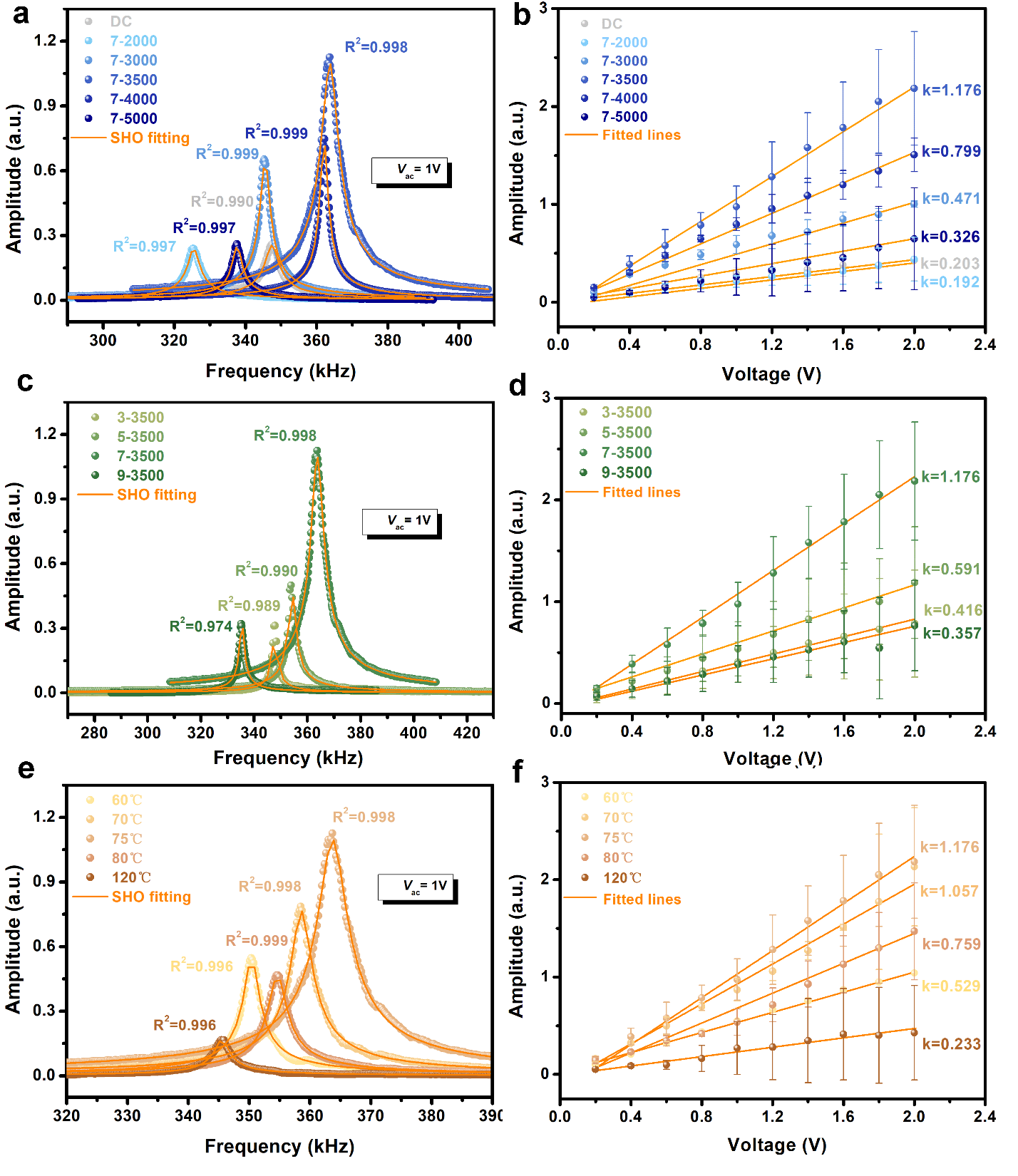


**Fig. S20 Piezoresponse of TMCM-CdBrCl_2_ films with varying**: **a, b)** ***U*** (T_S_=75℃)**, c, d)** ***H*** (T_S_=75℃) and **e, f) *T_S_***.


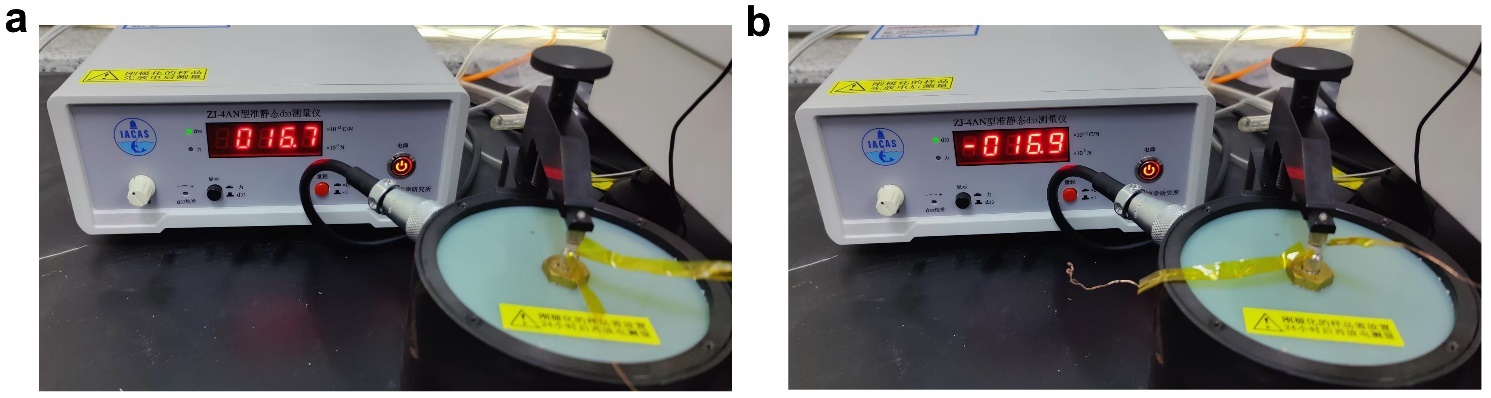


**Fig. S21 Photos of the d_33_ measurement on the TMCM-CdBrCl_2_ pellet using a d_33_ meter.**


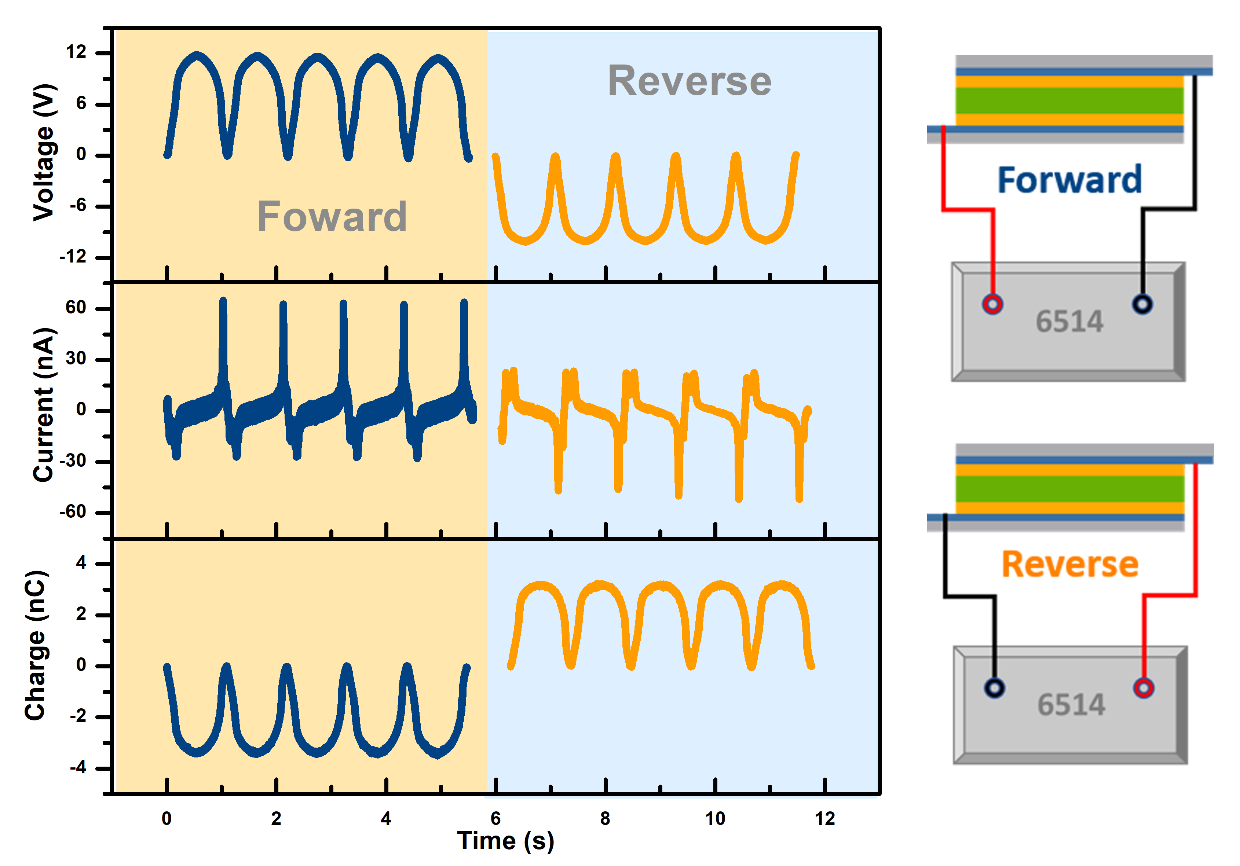


**Fig. S22 Forward and reverse output results of 7-3500@75℃ TMCM-CdBrCl_2_-based PENG.**


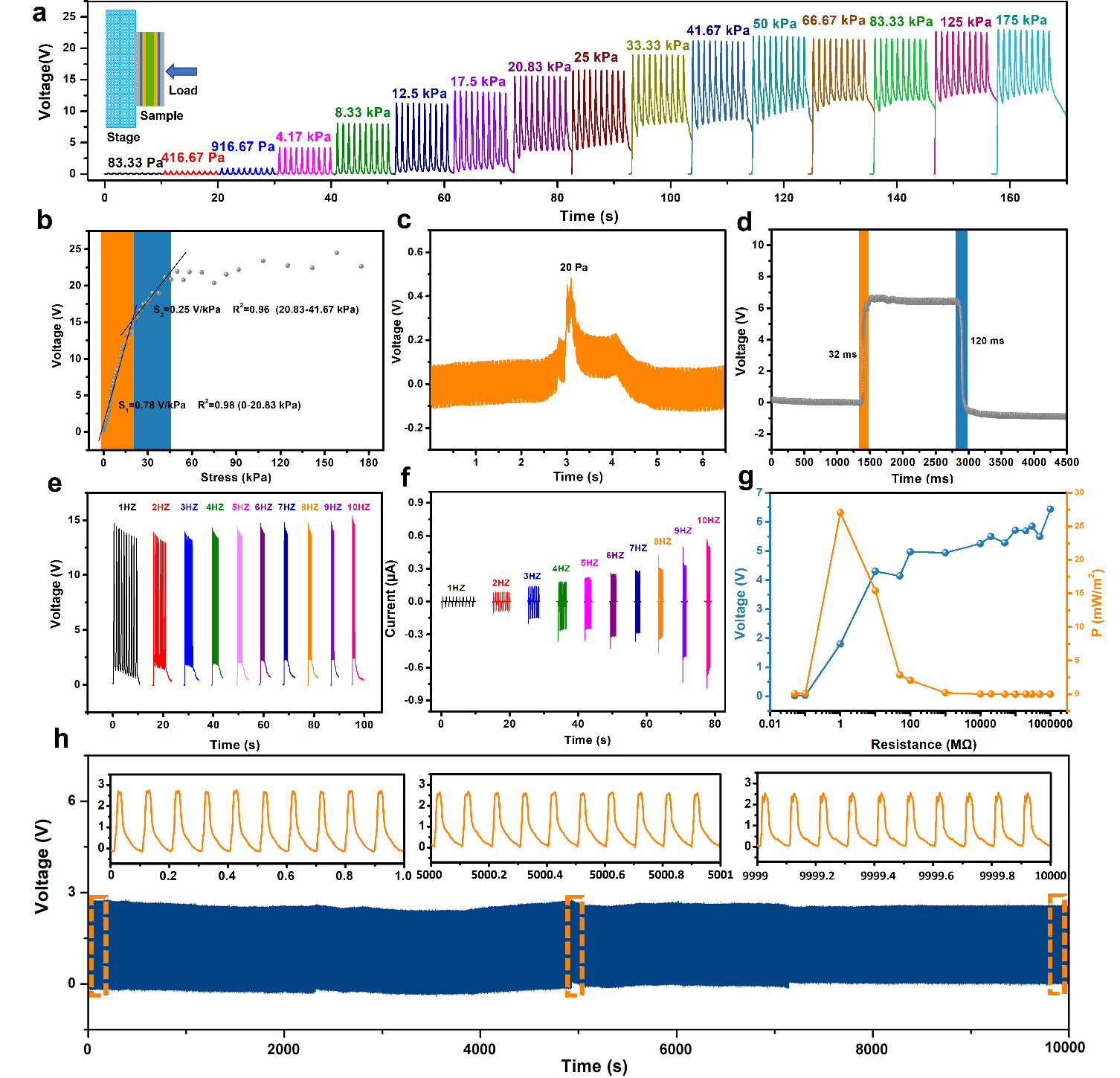


**Fig. S23 EHD-sprayed 5-4000@70℃ C_6_H_5_N(CH_3_)_3_CdBr_2_Cl_0.75_I_0.25_ film serving as a piezoelectric pressure sensor**: **a)** Stress-dependent voltage output characteristics; **b)** Calculated linear sensitivities in different stress ranges; **c)** Minimum stress detection limit; **d)** Response and recovery time; **e)** and **f)** Frequency-dependent voltage and current output results, respectively; **g)** Calculated output voltage and power density as a function of resistance; **h)** Output stability under 100,000 cycles (stress: 3.5 kPa; frequency: 10 Hz), in which the insets are the waveforms at the beginning, middle, and end of cycles.





**Fig. S24 The charge output of a 5-4000@70℃ C_6_H_5_N(CH_3_)_3_CdBr_2_Cl_0.75_I_0.25_-based piezoelectric sensor working under increasing frequencies.**


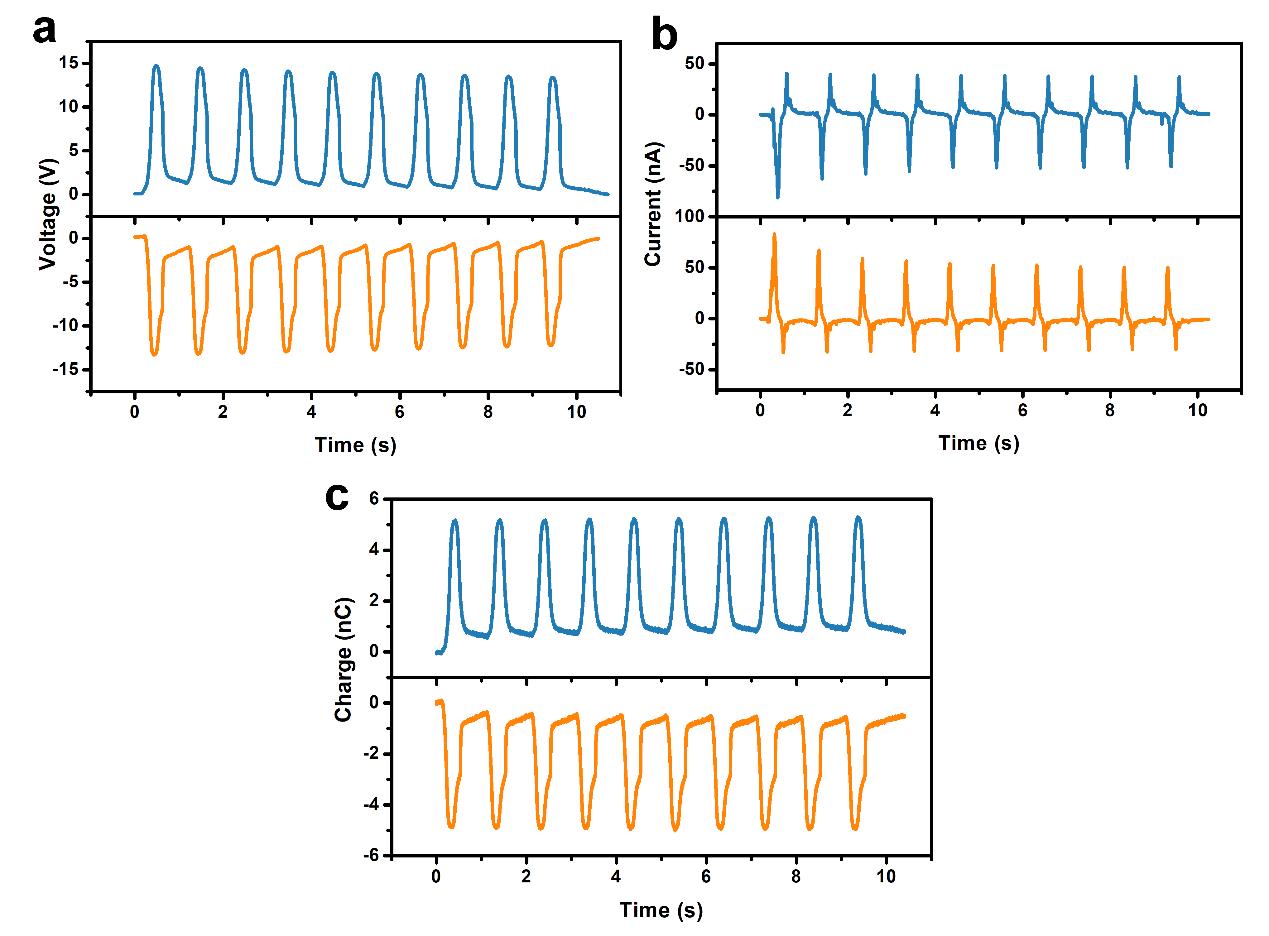


**Fig. S25 Polarization switching test results of 5-4000@70℃ C_6_H_5_N(CH_3_)_3_CdBr_2_Cl_0.75_I_0.25_-based piezoelectric sensor: a)** Output voltage, **b)** Short current, and **c)** Output charge.

**
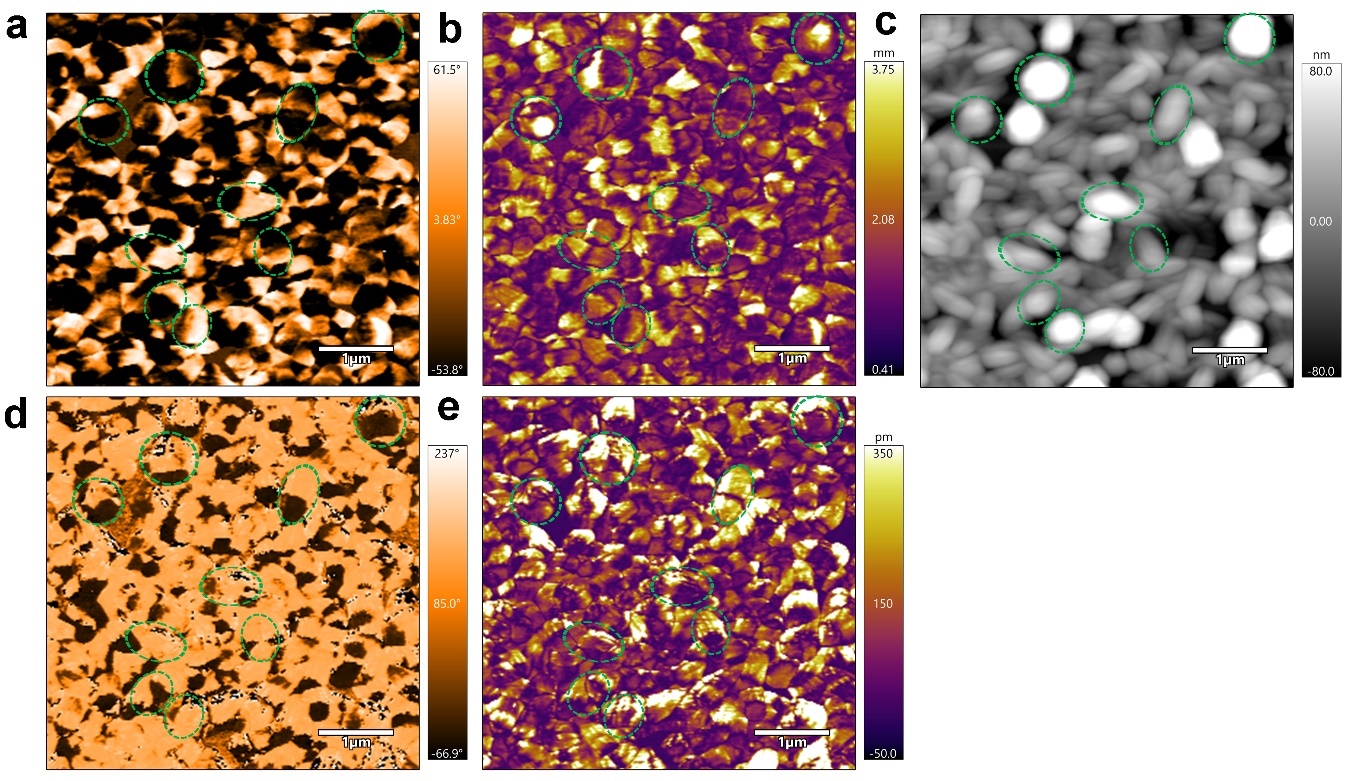
**

**Fig. S26 PFM images of EHD-sprayed TMCM-CdBrCl_2_ film stored for over one year**: **a)** and **b)** Lateral PFM phase and amplitude images, respectively; **c)** Topographic image; **d)** and **e)** Vertical PFM phase and amplitude images, respectively.


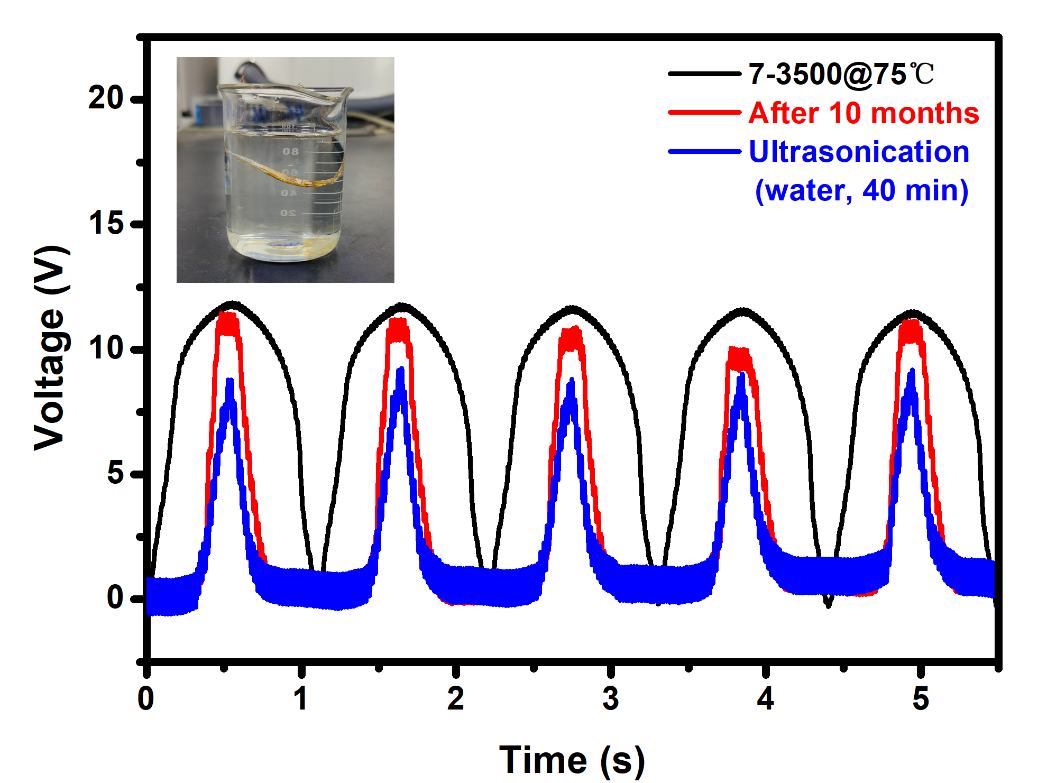


**Fig. S27 Environmental stability evaluation of TMCM-CdBrCl_2_ -based PENG under different conditions (insert is the picture of PENG immersing in water).**

**

**

**Fig. S28 FTIR spectra of prepared TMCM-CdBrCl_2_ film exposed to air for three days.**

**
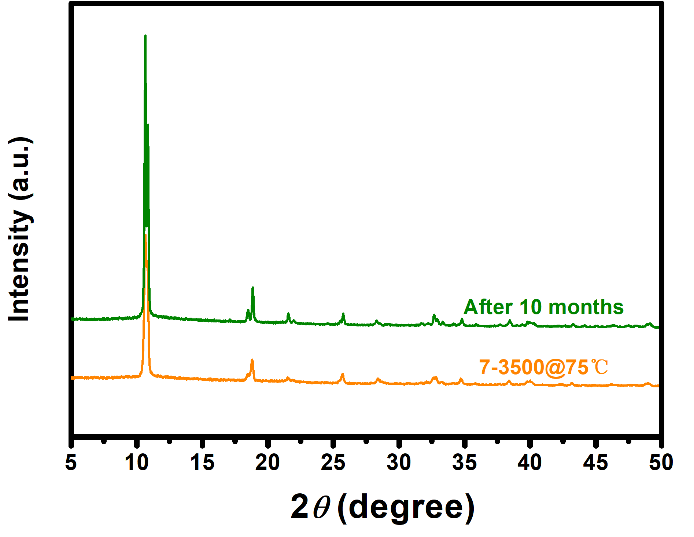
**

**Fig. S29 Environmental stability proof of PXRD results of** **TMCM-CdBrCl_2_ film measured at different times.**


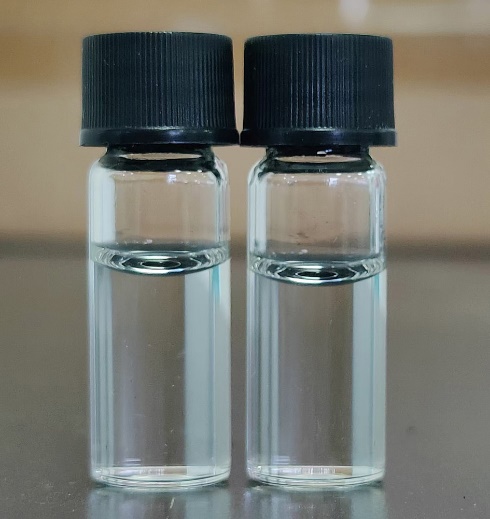


**Fig. S30 Picture of TMCM-CdBrCl_2_ (left) and C_6_H_5_N(CH_3_)_3_CdBr_2_Cl_0.75_I_0.25_** **(right) precursor solutions.**

**Table S1 Performance comparisons in various piezoelectric generators.**

| **Materials**  **type** | **Components (Substrate/poling condition)** | **Film deposition method**  **(Thickness)** | **Maximum output voltage/Sensitivity**  **(Strain/load, active area, frequency)** | **Durability (cycles)**  **(Strain/load, frequency)** | **Refs.** |
| --- | --- | --- | --- | --- | --- |
| Metal-free Organic Halide Perovskite Film | MDABCO-NH_4_I_3_  (PI/poled) | Spin-coating  (1.6 μm) | 15.9 V  (0.55%, 2.25 cm^2^, 1 Hz) | 5,000  (0.55%, 1 Hz) | [2](#_ENREF_2) |
| Inorganic Halide (Double) Perovskite Film | CsPbBr_3_  (ITO-PEN/poled) | Spin-coating & dip-coating  (545 nm) | 22.6 V  (0.67%, 0.16 cm^2^, 2.1 Hz) | 25,000  (0.75%, 2.1 Hz) | [3](#_ENREF_3) |
|  | CsPbBr_3_  (ITO-PET/poled) | Spin-coating  (260 nm) | 16.4 V  (1.67%, 5 cm^2^, 1 Hz) | 3,000  (1.67%, 1 Hz) | [4](#_ENREF_4) |
|  | CsSnI_3_  (ITO-PET/poled) | Spin coating  (4*354 nm) | 22.9 V  (NA, 6.25 cm^2^, NA) | NA | [5](#_ENREF_5) |
|  | Cs_2_AgBiBr_6_  (ITO-PET/NA) | Spin-coating  (NA) | 45 V  (25 kPa, 2.25 cm^2^, 2 Hz) | 1,000  (NA, NA) | [6](#_ENREF_6) |
| Organic-inorganic Hybrid Perovskites  (OIHPs) Film | MAPbI_3_  (ITO-PEN/poled) | Spin-coating & dip-coating  (486 nm) | 23.1 V  (0.47%, 14 cm^2^, 2.9 Hz) | 20,000  (0.47%, 2.9 Hz) | [7](#_ENREF_7) |
|  | MA_2_SnCl_6_  (ITO-PET/poled) | Spin-coating  (300 nm) | 0.29 V/N  (0.5-10 N, 1 cm^2^, 5 Hz) | 10,000  (50 N, 5 Hz) | [8](#_ENREF_8) |
|  | (FMTMA)PbCl_2_I  (Conductive tape/NA) | Spread-coating  (NA) | 4 V  (NA, 1 cm^2^, 3.2 Hz) | NA | [9](#_ENREF_9) |
|  | MAPb_0.93_Fe_0.07_I_3_  (ITO-PET/poled) | Spin-coating  (1 μm) | 7.29 V  (0.5 MPa, 1 cm^2^, 1 Hz) | 600  (0.5 MPa, 1 Hz) | [10](#_ENREF_10) |
|  | 4Cl-MAPbI_3_  (PI/poled) | Spin-coating  (350 nm) | 5.9 V  (0.5 MPa, 1 cm^2^, 2 Hz) | 2,000  (0.5MPa, 2 Hz) | [11](#_ENREF_11) |
|  | CH_3_NH_3_PbI_3_  (ITO-PET/poled) | Spin coating  (500 nm) | 2.7 V  (0.5 MPa, 1 cm^2^, 0.5 Hz) | NA | [12](#_ENREF_12) |
|  | TMCM-CdBrCl_2_  (ITO-PET/unpoled) | EHD-sprayed  (1.1 μm) | 11.93 V  (4.64%, 1.5 cm^2^, 1 Hz) | 200,000  (3.46%, 10 Hz) | This work |
|  | C_6_H_5_N(CH_3_)_3_CdBr_2_Cl_0.75_I_0.25_  (ITO-PET/unpoled) | EHD-sprayed  (108.7 nm) | 0.78 V kPa^−1^  (0-20.83 kPa, 1.5 cm^2^, 1 Hz) | 100,000  (3.5 kPa, 10 Hz) | This work |
| Perovskite Oxide Film | CaCu_3_Ti_4_O_12_  (PEN/poled) | RF magnetron sputtering  (497 nm) | 38.7 V  (0.77%, 3 cm^2^, 3.1 Hz) | 11,000  (0.77%, 2.9 Hz) | [13](#_ENREF_13) |
|  | PZT  (3M tape/poled) | Spin-coating  (700 nm) | 0.11 V degree^−1^  (15-90°, NA, 2 Hz)  1.76 V kPa^−1^  (0.32-1.6 kPa, NA, 2 Hz) | 12,000  (NA, 3 Hz) | [14](#_ENREF_14) |
|  | PZT  (ZRC/poled) | Spin-coating  (2 μm) | 105 V  (0.214%, 0.004 cm^2^, 4 Hz) | 15,000  (0.214%, 4 Hz) | [15](#_ENREF_15) |
|  | BaTiO_3_  (Mica/poled) | RF magnetron sputtering  (500 nm) | 0.5 V  (NA, 4 cm^2^, 0.3 Hz) | — | [16](#_ENREF_16) |
|  | Sm: PMN-PT  (Mica/poled) | Spin-coating  (850 nm) | 0.29 V kPa^−1^  (3.77-29.74 kPa, 0.49 cm^2^, 0.2 Hz) | 1,000  (NA, NA)  50,000  (NA, 10 Hz) | [17](#_ENREF_17) |
|  | PZT  (PET/poled) | Spin-coating  (100 nm) | 0.062 V kPa^−1^  (0-10 kPa, NA, NA) | 55,000  (10 kPa, 3 Hz) | [18](#_ENREF_18) |
| Composites | PUU/dabcoHReO_4_  (NA/NA) | Drop-coating  (320 μm) | 0.088 V kPa^−1^  (0.4-25.5 kPa, NA, 6 Hz) | 3,000  (25.5 kPa, 6 Hz) | [19](#_ENREF_19) |
|  | Cs_3_Bi_2_I_9_-PVDF  (NA/poled) | Drop-casting  (NA) | 0.044 V kPa^−1^  (0.5-10 kPa, 4 cm^2^, NA) | 18,000  (2.5 N, NA) | [20](#_ENREF_20) |
|  | [C(NH_2_)_3_]ClO_4_-PU  (NA/poled) | Soaking  (3 mm) | 0.17 V kPa^−1^  (0.003-5 kPa, 4 cm^2^, NA) | 3,000  (100 kPa, NA) | [21](#_ENREF_21) |
|  | ImClO_4_/BC  (NA/poled) | RT evaporation  (2 mm) | 0.004 V kPa^−1^  (0.2-31.25 kPa, 4 cm^2^, NA) | 2,000  (31.25 kPa, NA) | [22](#_ENREF_22) |
|  | [Hdabco]BF_4_@PVDF  (NA/unpoled) | Casting  (160 μm) | 0.4 V kPa^−1^  (1.6-12.8 kPa, 6.25 cm^2^, NA) | NA  (NA, NA) | [23](#_ENREF_23) |
|  | P(VDF-TrFE)-BaTiO_3_  (NA/unpoled) | DIW additive manufacturing  (NA) | 0.082 V kPa^−1^  (5-60 kPa, 1 cm^2^, 0.3 Hz) | 1,000  (30 kPa, 0.3 Hz) | [24](#_ENREF_24) |
|  | Ba_0.94_Sr_0.06_Sn_0.09_Ti_0.91_O_3_/PDMS  (PEN-ITO/poled) | Spin-coating  (NA) | 0.021 V kPa^−1^  (13.89-138.89 kPa, 4 cm^2^, 2 Hz) | 50,000  (NA, 3 Hz) | [25](#_ENREF_25) |

**Note:** “NA” denotes “Not Available”.

# References

1. Wu HS, Wei SM, Chen SW, Pan HC, Pan WP, Huang SM, et al. Meta‐free perovskite piezoelectric nanogenerators for human–machine interfaces and self‐powered electrical stimulation applications. Advanced Science 2022, 9(18): 2105974.

2. Kim DB, Jo KS, Park SJ, Cho YS. Contribution of anisotropic lattice‐strain to piezoelectricity and electromechanical power generation of flexible inorganic halide thin films. Advanced Energy Materials 2022, 12(11): 2103329.

3. Kim DB, Park KH, Cho YS. Origin of high piezoelectricity of inorganic halide perovskite thin films and their electromechanical energy-harvesting and physiological current-sensing characteristics. Energy & Environmental Science 2020, 13(7): 2077-2086.

4. Kim DB, Park KS, Park SJ, Cho YS. Microampere-level piezoelectric energy generation in Pb-free inorganic halide thin-film multilayers with Cu interlayers. Nano Energy 2022, 92: 106785.

5. Paul T, Sahoo A, Maiti S, Mandal S, Bhattacharjee S, Maity A, et al. Observation of piezoelectricity in a lead-free Cs_2_AgBiBr_6_ perovskite: a new entrant in the energy harvesting arena. Nanoscale 2024, 16(34): 16127-16139.

6. Kim DB, Jo KS, Park KS, Cho YS. Anion‐Dependent Polarization and Piezoelectric Power Generation in Hybrid Halide MAPbX_3_ (X= I, Br, and Cl) Thin Films with Out‐of‐Plane Structural Adjustments. Advanced Science 2023, 10(4): 2204462.

7. Ippili S, Kim JH, Jella V, Behera S, Vuong V-H, Jung J-S, et al. Halide double perovskite-based efficient mechanical energy harvester and storage devices for self-charging power unit. Nano Energy 2023, 107: 108148.

8. Zhang Z-X, Zhang H-Y, Zhang W, Chen X-G, Wang H, Xiong R-G. Organometallic-based hybrid perovskite piezoelectrics with a narrow band gap. Journal of the American Chemical Society 2020, 142(41): 17787-17794.

9. Ippili S, Jella V, Kim J, Hong S, Yoon S-G. Enhanced piezoelectric output performance via control of dielectrics in Fe^2+^-incorporated MAPbI_3_ perovskite thin films: Flexible piezoelectric generators. Nano Energy 2018, 49: 247-256.

10. Jella V, Ippili S, Yoon S-G. Halide (Cl/Br)-Incorporated Organic–Inorganic Metal Trihalide Perovskite Films: Study and Investigation of Dielectric Properties and Mechanical Energy Harvesting Performance. ACS Applied Electronic Materials 2020, 2(8): 2579-2590.

11. Kim Y-J, Dang T-V, Choi H-J, Park B-J, Eom J-H, Song H-A, et al. Piezoelectric properties of CH_3_NH_3_PbI_3_ perovskite thin films and their applications in piezoelectric generators. Journal of Materials Chemistry A 2016, 4(3): 756-763.

12. Han J, Park SH, Jung YS, Cho YS. High-performance piezoelectric energy harvesting in amorphous perovskite thin films deposited directly on a plastic substrate. Nature Communications 2024, 15(1): 4129.

13. Xu Q, Jia M, Zhou P, Zhang Y, Guo W, Zhao S, et al. High‐Performance Ultrasensitive Flexible Piezoelectric Thin Film Sensors via a Cost‐Effective Transfer Strategy. Advanced Functional Materials 2024: 2414211.

14. Liu Y, Ding L, Dai L, Gao X, Wu H, Wang S, et al. All‐Ceramic Flexible Piezoelectric Energy Harvester. Advanced functional materials 2022, 32(52): 2209297.

15. Hyeon DY, Park K-I. Piezoelectric flexible energy harvester based on BaTiO_3_ thin film enabled by exfoliating the mica substrate. Energy Technology 2019, 7(10): 1900638.

16. Lv P, Qian J, Yang C, Liu T, Wang Y, Wang D, et al. Flexible all-inorganic Sm-doped PMN-PT film with ultrahigh piezoelectric coefficient for mechanical energy harvesting, motion sensing, and human-machine interaction. Nano Energy 2022, 97: 107182.

17. Min S, Kim DH, Joe DJ, Kim BW, Jung YH, Lee JH, et al. Clinical validation of a wearable piezoelectric blood‐pressure sensor for continuous health monitoring. Advanced Materials 2023, 35(26): 2301627.

18. Li Q, Chen L, Guo M, Hu Z. Extremely stretchable and tough piezoelectric gels for artificial electronic skin. Advanced Materials Technologies 2022, 7(8): 2101371.

19. Mondal B, Mishra HK, Sengupta D, Kumar A, Babu A, Saini D, et al. Lead-free perovskite Cs_3_Bi_2_I_9_-derived electroactive PVDF composite-based piezoelectric nanogenerators for physiological signal monitoring and piezo-phototronic-aided strain modulated photodetectors. Langmuir 2022, 38(40): 12157-12172.

20. Li W, Li C, Zhang G, Li L, Huang K, Gong X, et al. Molecular ferroelectric‐based flexible sensors exhibiting supersensitivity and multimodal capability for detection. Advanced Materials 2021, 33(44): 2104107.

21. Lu J, Hu S, Li W, Wang X, Mo X, Gong X, et al. A biodegradable and recyclable piezoelectric sensor based on a molecular ferroelectric embedded in a bacterial cellulose hydrogel. ACS nano 2022, 16(3): 3744-3755.

22. Deswal S, Khandelwal G, Dahiya R. Molecular ferroelectric based biocompatible flexible piezoelectric pressure sensor. IEEE Sensors Letters 2023, 7(6): 1-4.

23. Nassar H, Khandelwal G, Chirila R, Karagiorgis X, Ginesi RE, Dahiya AS, et al. Fully 3D printed piezoelectric pressure sensor for dynamic tactile sensing. Additive Manufacturing 2023, 71: 103601.

24. Deng L, Deng W, Yang T, Tian G, Jin L, Zhang H, et al. Flexible lead-free piezoelectric Ba_0.94_Sr_0.06_Sn_0.09_Ti_0.91_O_3_/PDMS composite for self-powered human motion monitoring. Journal of Functional Biomaterials 2023, 14(1): 37.

1. Chen X-G, Tang Y-Y, Lv H-P, Song X-J, Peng H, Yu H*, et al.* Remarkable enhancement of piezoelectric performance by heavy halogen substitution in hybrid perovskite ferroelectrics. *Journal of the American Chemical Society* 2023, **145**(3)**:** 1936-1944.

2. Wu HS, Wei SM, Chen SW, Pan HC, Pan WP, Huang SM*, et al.* Metal‐free perovskite piezoelectric nanogenerators for human–machine interfaces and self‐powered electrical stimulation applications. *Advanced Science* 2022, **9**(18)**:** 2105974.

3. Kim DB, Jo KS, Park SJ, Cho YS. Contribution of anisotropic lattice‐strain to piezoelectricity and electromechanical power generation of flexible inorganic halide thin films. *Advanced Energy Materials* 2022, **12**(11)**:** 2103329.

4. Kim DB, Park KH, Cho YS. Origin of high piezoelectricity of inorganic halide perovskite thin films and their electromechanical energy-harvesting and physiological current-sensing characteristics. *Energy & Environmental Science* 2020, **13**(7)**:** 2077-2086.

5. Kim DB, Park KS, Park SJ, Cho YS. Microampere-level piezoelectric energy generation in Pb-free inorganic halide thin-film multilayers with Cu interlayers. *Nano Energy* 2022, **92:** 106785.

6. Paul T, Sahoo A, Maiti S, Mandal S, Bhattacharjee S, Maity A*, et al.* Observation of piezoelectricity in a lead-free Cs 2 AgBiBr 6 perovskite: a new entrant in the energy harvesting arena. *Nanoscale* 2024, **16**(34)**:** 16127-16139.

7. Kim DB, Jo KS, Park KS, Cho YS. Anion‐Dependent Polarization and Piezoelectric Power Generation in Hybrid Halide MAPbX3 (X= I, Br, and Cl) Thin Films with Out‐of‐Plane Structural Adjustments. *Advanced Science* 2023, **10**(4)**:** 2204462.

8. Ippili S, Kim JH, Jella V, Behera S, Vuong V-H, Jung J-S*, et al.* Halide double perovskite-based efficient mechanical energy harvester and storage devices for self-charging power unit. *Nano Energy* 2023, **107:** 108148.

9. Zhang Z-X, Zhang H-Y, Zhang W, Chen X-G, Wang H, Xiong R-G. Organometallic-based hybrid perovskite piezoelectrics with a narrow band gap. *Journal of the American Chemical Society* 2020, **142**(41)**:** 17787-17794.

10. Ippili S, Jella V, Kim J, Hong S, Yoon S-G. Enhanced piezoelectric output performance via control of dielectrics in Fe2+-incorporated MAPbI3 perovskite thin films: Flexible piezoelectric generators. *Nano Energy* 2018, **49:** 247-256.

11. Jella V, Ippili S, Yoon S-G. Halide (Cl/Br)-Incorporated Organic–Inorganic Metal Trihalide Perovskite Films: Study and Investigation of Dielectric Properties and Mechanical Energy Harvesting Performance. *ACS Applied Electronic Materials* 2020, **2**(8)**:** 2579-2590.

12. Kim Y-J, Dang T-V, Choi H-J, Park B-J, Eom J-H, Song H-A*, et al.* Piezoelectric properties of CH 3 NH 3 PbI 3 perovskite thin films and their applications in piezoelectric generators. *Journal of Materials Chemistry A* 2016, **4**(3)**:** 756-763.

13. Han J, Park SH, Jung YS, Cho YS. High-performance piezoelectric energy harvesting in amorphous perovskite thin films deposited directly on a plastic substrate. *Nature Communications* 2024, **15**(1)**:** 4129.

14. Xu Q, Jia M, Zhou P, Zhang Y, Guo W, Zhao S*, et al.* High‐Performance Ultrasensitive Flexible Piezoelectric Thin Film Sensors via a Cost‐Effective Transfer Strategy. *Advanced Functional Materials* 2024**:** 2414211.

15. Liu Y, Ding L, Dai L, Gao X, Wu H, Wang S*, et al.* All‐Ceramic Flexible Piezoelectric Energy Harvester. *Advanced functional materials* 2022, **32**(52)**:** 2209297.

16. Hyeon DY, Park K-I. Piezoelectric flexible energy harvester based on BaTiO3 thin film enabled by exfoliating the mica substrate. *Energy Technology* 2019, **7**(10)**:** 1900638.

17. Lv P, Qian J, Yang C, Liu T, Wang Y, Wang D*, et al.* Flexible all-inorganic Sm-doped PMN-PT film with ultrahigh piezoelectric coefficient for mechanical energy harvesting, motion sensing, and human-machine interaction. *Nano Energy* 2022, **97:** 107182.

18. Min S, Kim DH, Joe DJ, Kim BW, Jung YH, Lee JH*, et al.* Clinical validation of a wearable piezoelectric blood‐pressure sensor for continuous health monitoring. *Advanced Materials* 2023, **35**(26)**:** 2301627.

19. Li Q, Chen L, Guo M, Hu Z. Extremely stretchable and tough piezoelectric gels for artificial electronic skin. *Advanced Materials Technologies* 2022, **7**(8)**:** 2101371.

20. Mondal B, Mishra HK, Sengupta D, Kumar A, Babu A, Saini D*, et al.* Lead-free perovskite Cs3Bi2I9-derived electroactive PVDF composite-based piezoelectric nanogenerators for physiological signal monitoring and piezo-phototronic-aided strain modulated photodetectors. *Langmuir* 2022, **38**(40)**:** 12157-12172.

21. Li W, Li C, Zhang G, Li L, Huang K, Gong X*, et al.* Molecular ferroelectric‐based flexible sensors exhibiting supersensitivity and multimodal capability for detection. *Advanced Materials* 2021, **33**(44)**:** 2104107.

22. Lu J, Hu S, Li W, Wang X, Mo X, Gong X*, et al.* A biodegradable and recyclable piezoelectric sensor based on a molecular ferroelectric embedded in a bacterial cellulose hydrogel. *ACS nano* 2022, **16**(3)**:** 3744-3755.

23. Deswal S, Khandelwal G, Dahiya R. Molecular ferroelectric based biocompatible flexible piezoelectric pressure sensor. *IEEE Sensors Letters* 2023, **7**(6)**:** 1-4.

24. Nassar H, Khandelwal G, Chirila R, Karagiorgis X, Ginesi RE, Dahiya AS*, et al.* Fully 3D printed piezoelectric pressure sensor for dynamic tactile sensing. *Additive Manufacturing* 2023, **71:** 103601.

25. Deng L, Deng W, Yang T, Tian G, Jin L, Zhang H*, et al.* Flexible lead-free piezoelectric Ba0. 94Sr0. 06Sn0. 09Ti0. 91O3/PDMS composite for self-powered human motion monitoring. *Journal of Functional Biomaterials* 2023, **14**(1)**:** 37.
